# DNA barcoding, ecology and geography of the cryptic species of *Aneura pinguis* and their relationships with *Aneura maxima* and *Aneura mirabilis* (Metzgeriales, Marchantiophyta)

**DOI:** 10.1371/journal.pone.0188837

**Published:** 2017-12-05

**Authors:** Alina Bączkiewicz, Monika Szczecińska, Jakub Sawicki, Adam Stebel, Katarzyna Buczkowska

**Affiliations:** 1 Department of Genetics, Faculty of Biology, Adam Mickiewicz University, Poznań, Poland; 2 Department of Botany and Nature Protection, University of Warmia and Mazury in Olsztyn, Olsztyn, Poland; 3 Department of Pharmaceutical Botany, Medical University of Silesia in Katowice, Katowice, Poland; Indiana University Bloomington, UNITED STATES

## Abstract

*Aneura pinguis* is a thalloid liverwort species with broad geographical distribution. It is composed of cryptic species, however, the number of cryptic species within *A*. *pinguis* is not known. Five cpDNA regions (*matK*, *rbcL*, *rpoC1*, *trnH-psbA* and *trnL-trnF*) and the entire nuclear ITS region were studied in 130 samples of *A*. *pinguis* from different geographical regions. The relationships between the cryptic species of *A*. *pinguis*, *A*. *maxima* and *A*. *mirabilis* were analyzed. All of the examined samples were clustered into 10 clades corresponding to 10 cryptic species of *A*. *pinguis* (marked A to J). *Aneura mirabilis* and *A*. *maxima* were nested among different cryptic species of *A*. *pinguis*, which indicates that *A*. *pinguis* is a paraphyletic taxon. Subgroups were found in cryptic species A, B, C and E. As single barcodes, all tested DNA regions had 100% discriminant power and fulfilled DNA barcode criteria for species identification; however, the only combination detected in all subgroups was *trnL*-*trnF* with *trnH*-*psbA* or ITS2. The distances between cryptic species were 11- to 35-fold higher than intraspecific distances. In all analyzed DNA regions, the distances between most pairs of cryptic *A*. *pinguis* species were higher than between *A*. *maxima* and *A*. *mirabilis*. All cryptic species of *A*. *pinguis* clearly differed in their habitat preferences, which suggests that habitat adaptation could be the main driving force behind cryptic speciation within this taxon.

## Introduction

Taxonomy is a branch of biology concerned with the description, identification and classification of organisms and the phylogenetic relationships between them. The species is the fundamental unit in biology. The species concept and the delimitation of species have stirred much controversy since the early days of systematic biology [1]. Conflicting definitions of species have been proposed based on different criteria. According to Mayden [2], various aspects of lineage divergence arise at different times during the process of speciation [3]. The most popular definition of species is based on morphological differences [4, 5]. However, not all species can be identified based on morphological differences. In some cases, the accumulation of genetic and ecological differences is not correlated with the accumulation of morphological variations, this situation lead to appearance of cryptic species. Cryptic species are taxa which are characterized by distinctive genetic differences, different ecological preferences and the complete or nearly complete absence of morphological variations. For this reason, they cannot be identified based on the traditional morphological species concept [6, 7]. These species are difficult or impossible to identify based on their morphological traits, and they can be distinguished only with the use of biochemical or molecular methods [3, 8, 9]. DNA barcoding is a highly useful method for identifying taxonomically difficult species. The DNA barcoding concept is based on the presence of species-specific DNA sequences in one locus or multiple loci [10]. In recent years, quite a lot of new bryophyte species have been discovered by DNA barcoding [11–13]. These studies revealed that cryptic speciation in bryophytes is more common than previously thought.

*Aneura pinguis* (L.) Dumort. is a thalloid liverwort species with simple morphology and it is widespread in the Southern and Northern Hemispheres [14, 15]. The species is commonly found in diverse regions that extend from lowlands to high mountain zones, and it grows in various habitats, including calcareous rocks, humus, peat bogs, wet sands on lake shores and clay soils [16]. For over twenty years, it has been known that *A*. *pinguis* is a complex of cryptic species [9, 16–18]. Five cryptic species, provisionally named *A*. *pinguis* species A, B, C, D and E, have been identified to date. These species have been identified only in Europe, including four (A, B, C and E) in Central Europe and two (B and D) on the British Isles. The genetic differences among these species were as extensive as among related species, of other bryophytes and higher plants. Moreover there is no evidence to suggest recombination between these species [9, 16], i.e. they are species according to biological species concept. In cryptic species A, B and C, minor differences were found in morphological and anatomical features such as thallus and cell size, the thickness and number of cells in thallus cross-sections [19] and the size of oil bodies [20]. These differences are not sufficiently distinct and cannot be used as diagnostic features, however, they could support species identification. Wawrzyniak *et al*. [21] found qualitative differences in the composition of volatile compounds between cryptic species A, B, C and E of *A*. *pinguis*. Bączkiewicz *et al*. [22] reported differences in the environmental preferences of the analyzed species. The cryptic species of *A*. *pinguis* have never been formally described, and *A*. *pinguis* is still regarded as a taxonomically homogeneous species. However, the exact number of cryptic species within the entire geographical range of *A*. *pinguis* has not been unambiguously defined.

The main research aims of this study were to: i) analyze genetic differentiation within *A*. *pinguis*, ii) test the effectiveness of DNA barcoding (*matK*, *rbcL*, *rpoC1*, *trnH-psbA* and *trnL-trnF* and complete nuclear ITS) in the identification of cryptic species of *A*. *pinguis*, iii) analyze the evolutionary process of the *Aneura pinguis* complex, and the phylogenetic relationships between the cryptic species of *Aneura pinguis* and *A*. *mirabilis* and *A*. *maxima*.

## Materials and methods

### Plant material

Plant material consisted of 104 fresh samples and 26 herbarium specimens of *A*. *pinguis*, and 14 fresh samples of *A*. *maxima* from different geographical regions and different types of habitats (Tables 1 and 2 and S1 Table). The plants were initially identified based on morphological traits according to Schuster [23], and Buczkowska and Bączkiewicz [24]. Sequences from six DNA regions were newly generated for 70–143 specimens, depending on the region (GenBank accession numbers are listed in S1 Table). Several sequences of *rbcL*, *trnL-trnF* and ITS for the analyzed species were obtained from GenBank. The sequences of *A*. *mirabilis*, which was examined in this study for comparative purposes, *Pellia endiviifolia* (Dicks.) Dumort., *P*. *neesiana* (Gottsche) Limpr. and *Lobatiriccardia lobata* (Schiffn.) Furuki, selected as outgroups, were obtained from GenBank (Acc. No.: NC010359.1, AJ276490, AY507553.1, DQ986148.1).

**Table 1:** Number of studied samples from different geographical regions.

	No. of samples																
	*A*. *pinguis*																*A*. *maxima*
Regions	A1	A2	A3	B1	B2	B3	C1	C2	D	E1	E2	F	G	H	I	J	
Poland:																	
Wolin Island												3					
Western Pomerania				1		5	6			1			3		5		1
Warmia				1													1
Suwałki Lake District												1	2	2			
Wielkopolska				1			2							1*			2
Białowieża Forest																	3
Śląsk										1				1			
Tatra Mts	3	1	15		3		6			17		1					3
Beskidy Mts	4	3										2	1				1
Gorce Mts																	1
Pieniny Mts	2	1	2								1	2					
Góry Bialskie Mts												1					
Bieszczady Mts	2			2	4		1	3				6	1				2
Great Britain, Scotland, North Ebudes, Rum**									1								
Ireland, West Galway, Broadboy, Glencorbet **									1								
Romania																	1
Japan***																3	
New Zealand			1														
Canada										2							
U.S.A.												2					
Total	11	5	18	5	7	5	15	3	2	21	1	18	7	4	5	3	14

(leg.*P.Górski,.** D.G. Long, *** M. Itouga)

**Table 2:** Habitat characteristics of *Aneura pinguis* cryptic species and *A*. *maxima*.

Species/ cryptic species / lineages	No. of haplotypes	Habitat preferences
A1	3	humus over detritus flysch rocks or on humus over limestone rocks
A2	1	humus over detritus flysch rocks or on humus over limestone rocks
A3	4	humus over limestone rocks
B1	4	clay soil or on humus
B2	2	clay soil or on humus mixed with clay
B3	2	humus
C1	2	sandy soil or humus over limestone rocks or on humus or on rotten wood
C2	1	sandy soil
D	1	on wet flushed rock
E1	2	on rocks with leaking or flowing water
E2	1	on rocks with leaking water
F	4	clay soil and on humus mixed with clay
G	4	peat bog or peat covered lake shore, among *Sphagnum*
H	2	humus
I	2	peat covered lake shore, among *Sphagnum*
J	2	on wet flushed rock
*A*.*maxima*	5	in marsh situated on the river or stream banks

### Ethics statement

The samples of *A*. *pinguis* from the Tatra, Białowieża, Pieniny, Bieszczady and Wolin National Parks were collected by AB and KB with the permission given by the Ministry of Environment in Poland and the Directors of these National Parks. For the remaining locations specific permission was not required. *A*. *pinguis* is neither an endangered nor protected species.

### DNA extraction, PCR amplification and sequencing

Total genomic DNA was extracted from fresh material using the GeneJET Plant Genomic DNA Purification Mini Kit (Thermo Scientific) and from dried material using the Novabeads Plant DNA Kit (Novazym, Poland). The quality of isolated DNA was evaluated by electrophoresis in 0.8% agarose gel, and the concentration and purity of DNA samples were determined in the Epoch™ Multi-Volume Spectrophotometer System.

Six DNA regions, including five regions in the chloroplast genome (*matK*, *rbcL*, *rpoC1*, *trnH-psbA* and *trnL-trnF*) and the complete nuclear ITS region (ITS1-5.8S-ITS2) were analyzed. Standard barcode regions [25] were amplified for *rbcL* and *matK*. For *trnH*^GUG^*-psbA*, in addition to the spacer region, a fragment of the *psbA* gene was sequenced according to Bell et al. [26]. The *trnL-trnF* region contains the *trnL*^UAA^ gene (5’exon, intron and 3’exon) and the *trnL*^UAA^*-trnF*^*GAA*^ intergenic spacer [27]. Amplification and sequencing primers and PCR cycling conditions are given in S2 Table. PCR amplification was carried out according to the procedure described by Krawczyk et al. [28]. Purified PCR products of the studied DNA regions were sequenced in both directions using the same primers and the ABI BigDye 3.1 Terminator Cycle Kit (Applied Biosystems). The sequenced products were visualized using the ABI Prism 3130 Automated DNA Sequencer (Applied Biosystems). Bidirectional sequencing was applied to avoid sequencing errors.

### Data analysis

Chromatograms of DNA sequences were edited and assembled in Geneious R6 (Biomatters, USA). The assembled sequences were aligned in MEGA 6.06 [29] and Muscle [30] with default settings. Regions of ambiguous alignment and incomplete data were excluded from analysis. Seven individual DNA regions, five two-locus combinations and the combined dataset were evaluated in accordance with CBOL recommendations [25, 31] concerning potential barcode loci.

To illustrate differences between the examined specimens, neighbor joining trees were computed for individual and combined DNA regions. Separate analyses were performed for ITS1 and ITS2. Neighbor joining trees were generated based on the Kimura 2-parameter model [32] to enable comparison with other studies on DNA barcoding. Next, phylogenetic trees were generated by maximum parsimony (MP), maximum likelihood (ML) and Bayesian methods. The NJ, MP and ML analyses were carried out in MEGA 6.06, Bayesian inference in MrBayes 3.2 [33]. For both maximum likelihood and Bayesian analyses, the best model of evolution for the combined dataset (GTR+G+I) was determined using maximum likelihood model testing and the Bayesian Information Criterion (BIC) in MEGA 6.06, with four categories used for modeling the discrete gamma distribution.

Maximum parsimony analyses were performed with the following tree inference options: Tree-Bisection-Reconnection (TBR) as a search method with 10 initial trees (random-addition), search level 3, and the maximum number of 100 trees retained in each step. The confidence of clades within the inferred trees was evaluated by the bootstrap method with 1000 replicates.

Bayesian analysis was run on the combined dataset for four million generations with four simultaneous Markov chains. Model parameters and trees were sampled every 1000 generation. The first 25% of trees were discarded as burn-in. Bayesian posterior probability (BPP) confidence values generated from tree saved after this initial burn-in were used for estimatimation of clade support. Values ≥0.95% were regarded as significant.

The genetic distances for the pairs of sequences between and within the studied species were calculated using K2P and uncorrected p-distances to estimate evolutionary divergence and evaluate the effectiveness of the examined barcode loci. The mean, median, 90^th^ percentile and 95^th^ percentile were calculated for each tested locus for intra- and interspecific distances. The significance of differences between intraspecific and interspecific K2P distances was determined in the Mann–Whitney U test. The distribution of intraspecific and interspecific K2P distances for each examined locus was presented graphically to determine the presence of barcoding gaps and assess the effectiveness of barcode loci [10, 31, 34]. The presence of a classical barcoding gap was also checked by calculating the difference between the interspecific mean and the intraspecific mean and by verifying the 10-fold rule proposed by Hebert et al. [35]. *Aneura mirabilis* was represented by one sample and was excluded from barcoding gap analysis.

The Automatic Barcode Gap Discovery (ABGD) software was used to split the examined specimens of *A*. *pinguis* into candidate cryptic species based on pairwise distances by detecting differences between the intraspecific and interspecific variation (i.e. barcoding gap) without a priori species hypothesis. The method automatically find the distance where the barcode gap is located and can be used even when the two distributions (intraspecific and interspecific) overlap to partition the data set into candidate species [36]. ABGD analyses were performed on a web interface (http://www.abi.snv.jussieu.fr/public/abgd/abgdweb.html) with the use of all available distance metrics: JC69 [37], K2P and the uncorrected p-distance. Default values of P (Pmin = 0.001, Pmax = 0.1) and relative gap width X = 1.5 were used, with the exception of *rpoC1* where relative gap width was X = 1.2.

Haplotype networks with the MJ option (median joining; [38]) were calculated to examine variation and the relationships between the studied species. The MP option [39] was applied to identify redundant median vectors and links. Haplotype networks were developed in Network 5.0 (Fluxus Technology). The geographic location of each specimen carrying a given haplotype was coded to illustrate its distribution range. The pairwise homoplasy test (PHI) implemented in Splits-Tree 4 [40] was applied to detect possible recombination events in nrITS sequences between cryptic species of *A*. *pinguis*.

## Results

### Sequencing success and the characteristics of sequences

In all examined samples, high-quality DNA sequences were obtained for *matK*, *trnL-trnF*, ITS1 and ITS2. Regions *rbcL*, *rpoC1* and *trnH-psbA* were amplified with 100% efficiency, but high-quality sequences were obtained only in 85.6%, 53.4% and 74.6% of the analyzed samples, respectively. Sequences of satisfactory quality were used in alignment analysis. A total of 3569 bp were aligned in the examined chloroplast regions in genus *Aneura*, including 509 variable sites and 460 parsimony informative sites. The nuclear ITS1-5.8S-ITS2 region was composed of 753 bp, including 207 variable sites and 195 parsimony informative sites. The lengths of the analyzed DNA sequences with variable and parsimony informative sites for the examined plastid loci and separately for nuclear loci ITS1 and ITS2 are given in Table 3. The most parsimony informative loci were ITS1 (31.15%) and ITS2 (30.50%), followed by plastid loci *trnL-trnF* (14.92%), *matK* (14.32%) and *trnH-psbA* (14.13%), whereas *rbcL* was the least parsimony informative locus (8.27%).

**Table 3:** The length of examined DNA regions in the studied species of *Aneura*.

	*rbcL*	*matK*	*rpoC*1	*trnL-F*	*trnH-pabA*	ITS1	ITS2	ITS
*A*. *pinguis*								
A1	617	817	765	543	821	345–346	254	743
A2	617	817	765	540	821	346	254	743
A3	617	817	765	543	817	346	254	742–743
B1	617	817	765	545	794	349–350	249	741–742
B2	617	817	765	545	794	349	249	741
B3	617	817	765	545	796–798	349	249	741
C1	617	817	765	543	803	348	249	740
C2	617	817	765	543	796	347	249	739
D	617	817	765	539	794	345	255	743
E1	617	817	765	543	801	345	254	742
E2	617	817	765	543	801	345	254	742
F	617	817	765	543	802	348	249	740
G	617	817	765	543	794	341	254	738
H	617	817	765	543	793	348	257	748
I	617	817	765	543	805	347	255	745
J	617	817	765	543	790	345	256	744
*A*. *maxima*	617	817	765	543	799	347	254	744
*A*. *mirabilis*	616	817	765	552	817	347	254	744
Alignment length	617	817	765	555	828	351	259	753
Conserved sites	556	687	663	457	691	235	174	546
Variable sites (V)	61	130	102	89	137	115	85	207
Parsi-info sites (P)	51	117	87	82	117	110	79	195
% Parsi-info	8.27	14.32	11.37	14.77	14.13	31.15	30.50	25.90%
Singleton sites (S)	10	13	15	7	20	5	6	12

### DNA barcode variation in *A*. *pinguis*

Nucleotide diversity in the analyzed DNA regions of *A*. *pinguis* was determined at 2.15% to 10.32% in the K2P model. The nuclear region ITS1 was most variable. Nuclear regions ITS1 and ITS2 were more variable than chloroplast genome sequences, and average variation reached 9.49% and 3.52%, respectively. The most diverse chloroplast locus was *matK*, and the least diverse locus was *rbcL* (Table 4). The variations in the corresponding DNA regions of *A*. *maxima* were 80-fold smaller on average than in *A*. *pinguis*, and were determined at 0% in *rpoC1* and *trnH-psbA* to 0.20% in ITS2. The average variation in *A*. *maxima* was 0.07%, and it reached 0.09% in barcode locus *rbcL* and 0.04% in *matK* (Table 4). Uncorrected p-distances were somewhat lower than K2P in all analyzed DNA regions.

**Table 4:** Genetic differentiation (%) in the examined DNA regions of *Aneura pinguis* and *A*. *maxima* based on K2P model of nucleotide substitution.

Species	*matK*	*rbcL*	*rpoC*1	*trnL-trnF*	*trnH-psbA*	combined cp	ITS1	ITS2	ITS	combined data set
*A*. *pinguis*	4.24	2.15	3.46	3.94	3.82	3.55	10.32	8.66	7.72	4.23
*A*. *maxima*	0.04	0.09	0.00	0.08	0.00	0.04	0.08	0.20	0.11	0.06

### Identification of cryptic species within *A*. *pinguis*

**Fig 1:**
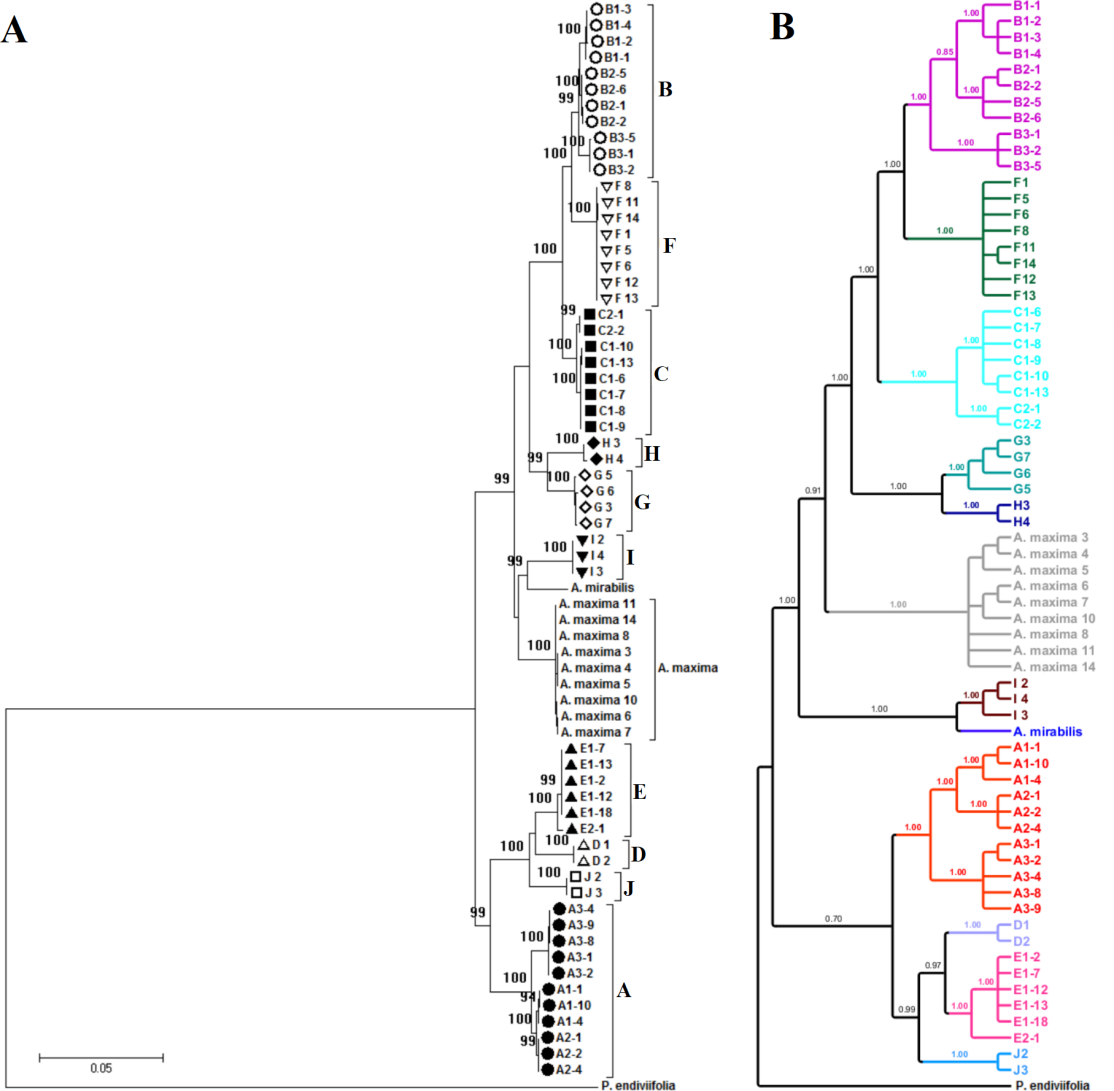
**Phylogeny of *Aneura pinguis* cryptic species obtained by: Maximum likelihood (A) and Bayesian (B) methods** based on a combined dataset. *Aneura maxima* and *A*. *mirabilis* were used for comparison. *Pellia endiviifolia* was used as an outgroup. Only the accessions with the sequences obtained for all loci were included in the analysis. The maximum likelihood tree with highest log likelihood (-15136.92) and Bootstrap values above 85% is shown. Bayesian posterior probabilities > 0.95redibility are given above the branches.

The cryptic species of the *A*. *pinguis* complex were identified in phylogenetic analyses in the first stage of the study. The analyses conducted with the use of NJ and MP methods revealed stable topology and the complex structure of *A*. *pinguis*. Maximum parsimony analyses of combined plastid loci and the nuclear ITS locus produced trees with identical topology to NJ trees. The two datasets could be combined due to the absence of differences in the topology of plastid and ITS trees. The ML analysis of the combined dataset resulted in a single optimal topology (-ln = 15136.9224) and revealed two major clades differentiated the analyzed *Aneura* species and resolved *A*. *pinguis* as a paraphyletic species. The same topologies were obtained from Bayesian interference of phylogeny, maximum parimony and neighbor joining analyses (Fig 1 and S1 Fig). The first major clade contained four clades of *A*. *pinguis*, and the second clade consisted of six clades of *A*. *pinguis* as well as clades of *A*. *maxima* and *A*. *mirabilis*. All of the examined samples of *A*. *pinguis* were clustered into 10 clades (marked A to J) with high bootstrap values (BS 99–100%, BPP>0.95) (Fig 1). Three of the tested loci (*matK*, *rbcL*, *trnH-psbA*) and two-gene combination cluster of *A*. *pinguis* into the same 10 clades with BS>80%. The remaining loci did not correctly distinguish species B (BS support <50%) but divided it into two or three clades with high BS value (S2 Fig).

In the K2P model, genetic divergence between the 10 cryptic species of *A*. *pinguis* ranged from 1.45% to 7.41% for the combined dataset. The lowest genetic divergence (1.45%) was found for species pairs B-C and B-F. The highest genetic distances were observed in species pairs D-F, F-J and E-F at 7.41%, 7.38% and 7.26%, respectively (Table 5). Of the two loci considered as core barcodes for plants (*rbcL* and *matK*), greater differences between the examined cryptic species occur in the *matK* region, but both regions support discrimination between all cryptic species. In *matK*, genetic difference was highest in species pair F-J (8.30%) and lowest in pair G-H (1.24%). In *rbcL*, genetic difference was highest in pair F-E (4.29%) and lowest in B-C (0.78%). In combined plastid loci, genetic divergence ranged from 1.22% to 6.38%, whereas in the ITS–from 1.58% to 12.97%. In nuclear regions, genetic differences between cryptic species ranged from 1.77% to 17.97% in ITS1 (lowest for the species pair B-F and highest for E-F) and from 1.20% to 15.43% in ITS2 (lowest for the species pair C-F and highest for E-H) (S3 Table). Uncorrected p-distances between the examined cryptic species were somewhat lower than K2P. Statistically significant evidence for recombination between clades in the nrITS region was not found in the PHI test (p = 0.3275).

**Table 5:** Average genetic divergences (%) for *Aneura pinguis* (A-J) cryptic species, *A*. *maxima* and *A*. *mirabilis*, based on the combined data set K2P (below diagonal) and uncorrected p-distance (above diagonal).

	A	B	C	D	E	F	G	H	I	J	*A*. *maxima*	*A*. *mirabilis*
A	***	5.72	5.54	4.55	4.34	6.06	5.05	5.76	5.13	4.42	4.98	5.26
B	6.00	***	1.43	6.49	6.31	1.43	3.53	3.56	4.21	6.60	3.59	4.13
C	5.81	1.45	***	6.39	6.13	1.90	3.48	3.69	4.00	6.44	3.28	4.01
D	4.72	6.84	6.74	***	2.57	7.00	5.84	6.22	5.68	3.17	5.39	5.91
E	4.50	6.64	6.44	2.62	***	6.87	5.48	5.85	5.47	2.55	5.01	5.50
F	6.37	1.45	1.93	7.41	7.26	***	3.96	4.15	4.62	6.97	3.98	4.62
G	5.27	3.63	3.58	6.13	5.73	4.09	***	2.43	3.69	5.71	3.36	3.93
H	6.05	3.66	3.80	6.54	6.13	4.29	2.47	***	4.16	6.26	3.62	4.10
I	5.35	4.35	4.13	5.94	5.72	4.79	3.79	4.30	***	5.77	3.35	3.17
J	4.59	6.97	6.79	3.25	2.60	7.38	5.98	6.59	6.05	***	5.57	5.80
*A*. *maxima*	5.19	3.70	3.37	5.64	5.22	4.11	3.45	3.73	3.44	5.84	***	3.04
*A*. *mirabilis*	5.50	4.27	4.14	6.21	5.75	4.80	4.06	4.23	3.25	6.09	3.11	***

### Differentiation within cryptic species

All examined DNA regions within the cryptic species of *A*. *pinguis* showed intraspecific variation. The highest intraspecific variation was detected in species A and B. Sequence diversity in species A was 0.543% in the combined dataset (0.375% in plastid and 1.362% in nrITS sequences) (Table 6). In individual loci, sequence diversity ranged from 0.106% to 1.817%, and it was lowest in *matK* and highest in ITS1. Sequence diversity in cryptic species B was 0.482% (0.397% in plastid and 0.886 in nrITS sequences).

**Table 6:** K2P (%) genetic variation in the DNA sequences of studied groups of *Aneura pinguis*.

	combined cp loci			ITS			combined data set		
	cryptic species	groups		cryptic species	groups		cryptic species	groups	
	0.375	A1	0.019	1.362	A1	0.124	0.543	A1	0.031
A		A2	0.000		A2	0.000		A2	0.000
		A3	0.028		A3	0.087		A3	0.024
	0.397	B1	0.000	0.886	B1	0.191	0.482	B1	0.047
B		B2	0.000		B2	0.091		B2	0.016
		B3	0.019		B3	0.000		B3	0.016
C	0.110	C1	0.000	0.225	C1	0.062	0.141	C1	0.013
		C2	0.000		C2	0.000		C2	0.000
D	0.000			0.000			0.000		
E	0.104	E1	0.000	0.135	E1	0.064	0.137	E1	0.016
		E	n/c		E2	n/c		E2	n/c
F	0.025			0.056			0.024		
G	0.076			0.165			0.098		
H	0.257			0.000			0.212		
I	0.019			0.000			0.016		
J	0.000			0.182			0.024		

In cryptic species A and B, three well-supported (BSP 95–100%) monophyletic lineages were identified in the MP tree (Fig 1). These lineages could not be classified as separate species based on the differences in their DNA sequences, and they were regarded as different groups of cryptic species. The groups of cryptic species A were labeled A1, A2, A3, and the groups of cryptic species B–B1, B2, B3.

Two well-supported evolutionary lineages were also identified in cryptic species C and E which were labeled C1, C2 and E1, E2, respectively (Fig 1). Genetic distances based on the combined dataset ranged from 0.188% to 0.944% between the lineages of cryptic species A, from 0.377 to 0.942% between the lineages of cryptic species B, and between the lineages of species C and E were 0.314% and 0.377%, respectively. In the nuclear ITS region, the distances were greater and ranged from 0.205% to 2.625% between the lineages of cryptic species A, from 0.948% to 1.573% between the lineages of cryptic species B, and 0.622% 0.641 between the lineages of species C and E, respectively (S4 Table).

### Haplotype network

**Fig 2:**
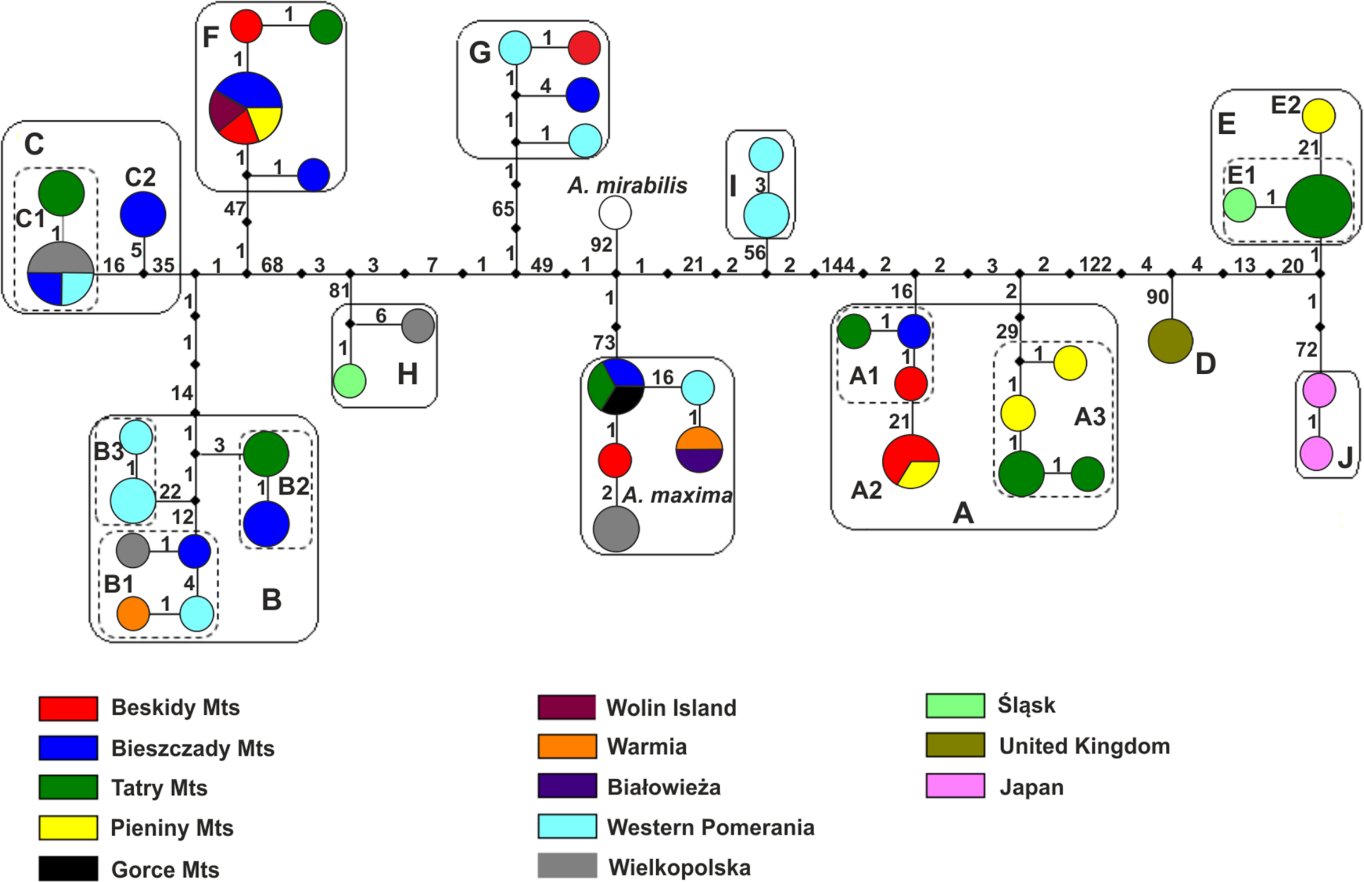
A haplotype network of the studied *Aneura* samples based on the combined dataset. Colored circles represent haplotypes. Colors represent the geographic origin of the specimens. Diameters denote the number of specimens carrying a particular haplotype, the smallest circle represents a single individual, and the largest circle represents five individuals. Black squares represent median vectors and figures–the number of mutation steps.

Thirty-seven haplotypes of *A*. *pinguis* were identified in the combined dataset (Table 2, Fig 2). The number of haplotypes ranged from 17 to 22 in individual chloroplast loci, and it was determined at 24 in ITS2 and 29 in ITS1. Based on the combined dataset, haplotypes were divided into 10 separate clades (A-J) corresponding to the cryptic species identified within *A*. *pinguis* using the phylogenetic tree. Haplotypes of *A*. *maxima* and *A*. *mirabilis* formed two separate clades. Individual cryptic species of *A*. *pinguis* harbored one to eight different haplotypes. The highest number of haplotypes was noted in species A and B which can be divided into three groups corresponding to lineages A1, A2, A3, and B1, B2, B3, separated by 10–52 mutation steps. Two haplotype groups separated by 21 and 15 mutation steps, respectively, were also found in cryptic species C and E (Fig 2).

### Intraspecific and interspecific distances and the barcoding gap

**Fig 3:**
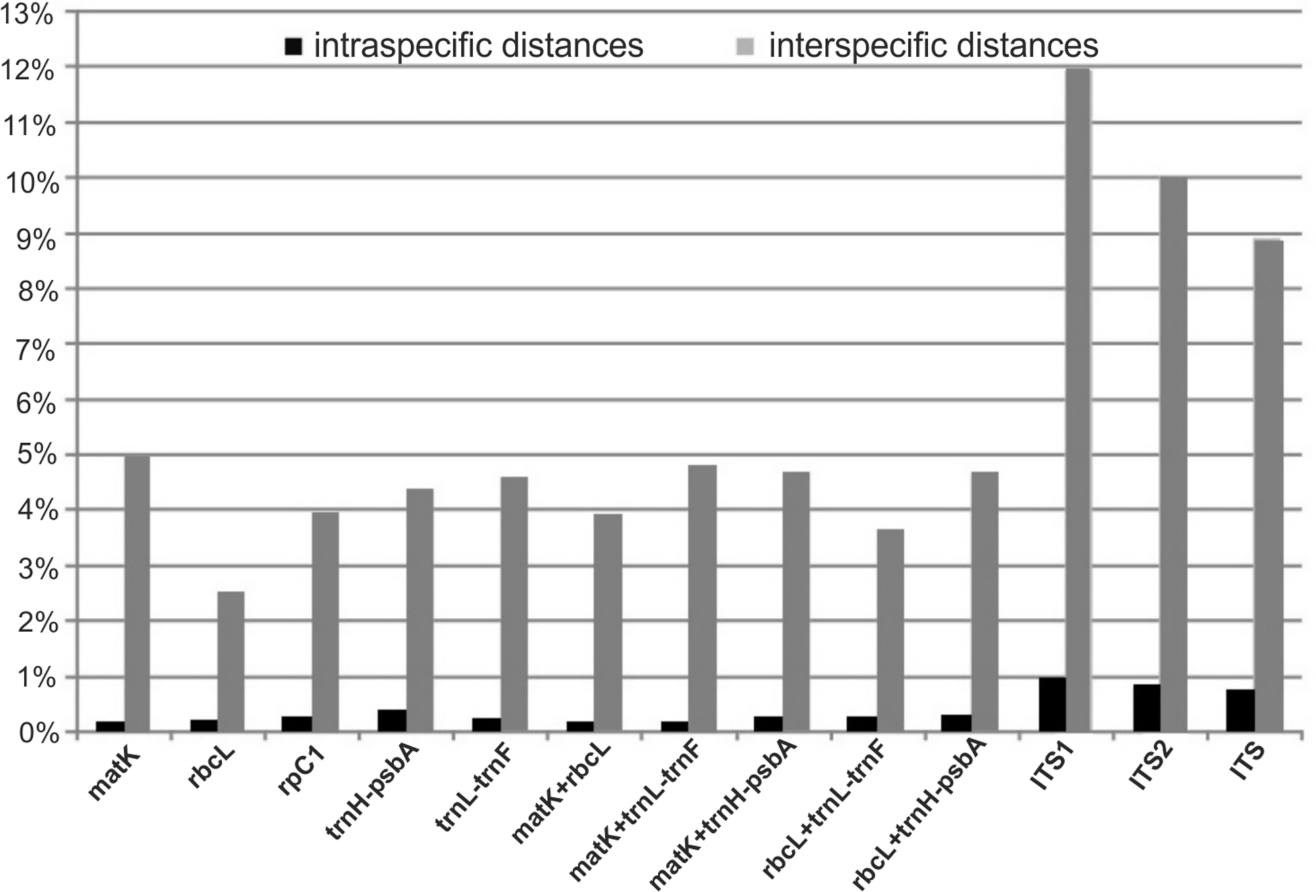
Mean intraspecific and interspecific K2P distances of individual loci and their combinations in *Aneura pinguis*.

**Fig 4:**
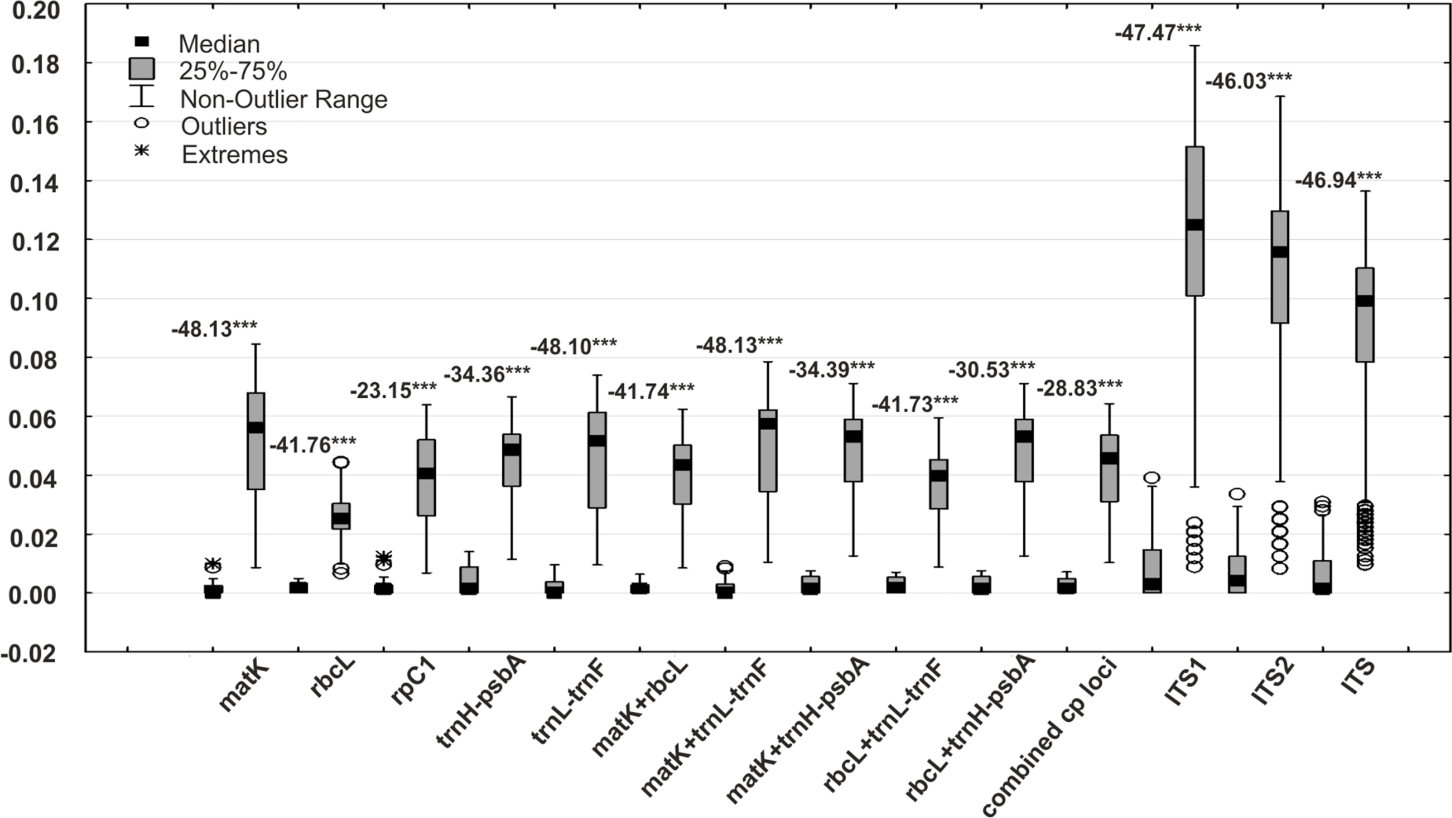
The ranges of intraspecific and interspecific K2P distances in *Aneura pinguis*. The ranges of intraspecific (the first box) and interspecific (the second box) distances for individual studied DNA regions and their combinations with the results of the Mann-Whitney test were compared.

Intraspecific and interspecific variation in the analyzed loci was calculated for the set of the cryptic species which were identified within *A*. *pinguis* based on the NJ tree. The greatest mean interspecific distances were found for nuclear loci (ITS1 = 11.94%, ITS2 = 10.02%), and the smallest distance (2.51%) was determined in the *rbcL* barcode locus (Fig 3). In plastid loci, the greatest (4.96%) mean interspecific variation was found in the *matK* barcode locus, and it was the highest difference in the analyzed plastid regions (Table 7). Uncorrected p-distances were somewhat lower than K2P in all of the analyzed DNA regions. The Mann–Whitney test revealed significant differences between the mean values of intraspecific and interspecific distances for each examined DNA region (Fig 4). The ranges of intraspecific and interspecific distances, means and medians for the tested loci and their combinations are given in Table 7.

**Table 7:** Parameters of intra- and interspecific variation of *Aneura pinguis* based on K2P (%) model of nucleotide substitution.

DNA region		N	Mean	Mean	Median	Min	Max	Overlap^1^	Percentile	Percentile	Overla^2^	Percentile	Percentile	Overla^3^
				inter-										
				/mean intraspecific					10%	90%		5%	95%	
	intra	792	0.14		0.05	0.00	0.99		0.00	0.25		0.00	0.86	
*matK*	inter	4564	4.96	35×	5.60	0.86	8.45	0.13	1.98	6.95	0	1.61	7.21	0
	intra	576	0.18		0.16	0.00	0.49		0.00	0.33		0.00	0.49	
*rbcL*	inter	3165	2.51	14×	2.52	0.66	4.44	0	1.16	3.37	0	0.82	3.90	0
	intra	148	0.25		0.14	0.00	1.23		0.00	1.08		0.00	1.09	
*rpoC1*	inter	1077	3.92	16×	4.04	0.68	6.39	0.55	1.49	5.89	0	1.09	6.09	0
	intra	390	0.38		0.13	0.00	1.40		0.00	1.02		0.00	1.15	
*trnH-psbA*	inter	2536	4.35	11×	4.85	1.14	6.66	0.26	2.31	5.60	0	1.66	5.79	0
	intra	792	0.21		0.08	0.00	0.95		0.00	0.76		0.00	0.76	
*trnL-trnF*	inter	4564	4.58	22×	5.16	0.95	7.39	0	2.30	6.35	0	1.34	7.16	0
	intra	576	0.16		0.14	0.00	0.63		0.00	0.28		0.00	0.63	
*matK+rbcL*	inter	3165	3.91	24×	4.34	0.85	6.24	0	1.77	5.17	0	1.41	6.33	0
	intra	792	0.16		0.07	0.00	0.90		0.00	0.37		0.00	0.75	
*matK+trnL-trnF*	inter	4564	4.81	30×	5.74	1.05	7.85	0	1.96	6.62	0	1.58	7.11	0
	intra	390	0.25		0.12	0.00	0.75		0.00	0.62		0.00	0.69	
*matK+trnH-psbA*	inter	2536	4.68	18×	5.30	1.25	7.11	0	2.35	6.17	0	1.70	6.62	0
	intra	576	0.24		0.17	0.00	0.70		0.00	0.61		0.00	0.61	
*rbcL+trnL-trnF*	inter	3165	3.63	15×	3.97	0.88	5.95	0	1.86	4.90	0	1.23	5.18	0
	intra	390	0.25		0.12	0.00	0.75		0.00	0.62		0.00	0.69	
*rbcL+trnH-psbA*	inter	2536	4.68	12×	5.30	1.25	7.11	0	2.02	6.34	0	1.70	6.62	0
	intra	338	0.24		0.14	0.00	0.71		0.00	0.69		0.00	0.69	
combined cp loci	inter	1542	4.14	17×	4.56	1.03	6.40	0	1.81	5.60	0	1.38	6.15	0
	intra	792	0.94		0.29	0.00	3.91		0.00	3.29		0.00	3.60	
ITS1	inter	4564	11.94	13×	12.50	0.88	18.59	3.03	4.58	16.58	0	3.91	17.72	0
	intra	792	0.80		0.41	0.00	3.35		0.00	2.49		0.00	2.50	
ITS2	inter	4564	10.02	12×	11.57	0.82	16.85	2.53	2.49	13.94	0	1.23	14.62	0.0127
	intra	792	0.74		0.14	0.00	3.08		0.00	2.51		0.00	2.65	
ITS	inter	4564	8.92	12×	9.91	0.96	13.64	2.12	2.80	12.11	0	2.37	12.79	0.0028
	intra	338	0.31		0.09	0.00	1.04		0.00	0.97		0.00	0.99	
combined data set	inter	1542	4.78	15.4×	5.12	1.28	7.45	0	1.96	6.60	0	1.47	6.88	0

Note: Overlap^1^ = Maximum of intraspecific—Minimum of interspecific distances; Overlap^2^ = 90% of intraspecific—10% of interspecific distances; Overlap^3^ = 95% of intraspecific—5% of interspecific distances.

A barcoding gap was detected in *rbcL*, *trnL-trnF*, in all two-gene combinations and in all combined chloroplast loci, which supported 100% discrimination of individuals. In *matK*, *rpoC1* and *trnH-psbA*, certain overlaps were noted in the ranges of intraspecific and interspecific distances (Fig 4, S3 Fig). A clear barcoding gap was not determined in ITS1, ITS2 or in the entire ITS. However, mean interspecific distances were 11- to 35-fold higher than mean intraspecific distances. The greatest differences between intraspecific and interspecific means were noted in *matK* (35-fold) and *trnL-trnF* (22-fold), and the smallest differences were observed in *trnH-psbA* (11-fold) and ITS2 (12-fold). Median values (the preferred statistics for non-normal distribution) were even higher, and up to 112-fold differences were noted in *matK* (Table 7). For all loci, the overlap between the largest intraspecific distance and smallest interspecific distance did not occur at the 90th intraspecific percentile and the 10th interspecific percentile, and, with the exception of ITS2 and entire ITS, even at the 95 and 5th percentile.

**Fig 5:**
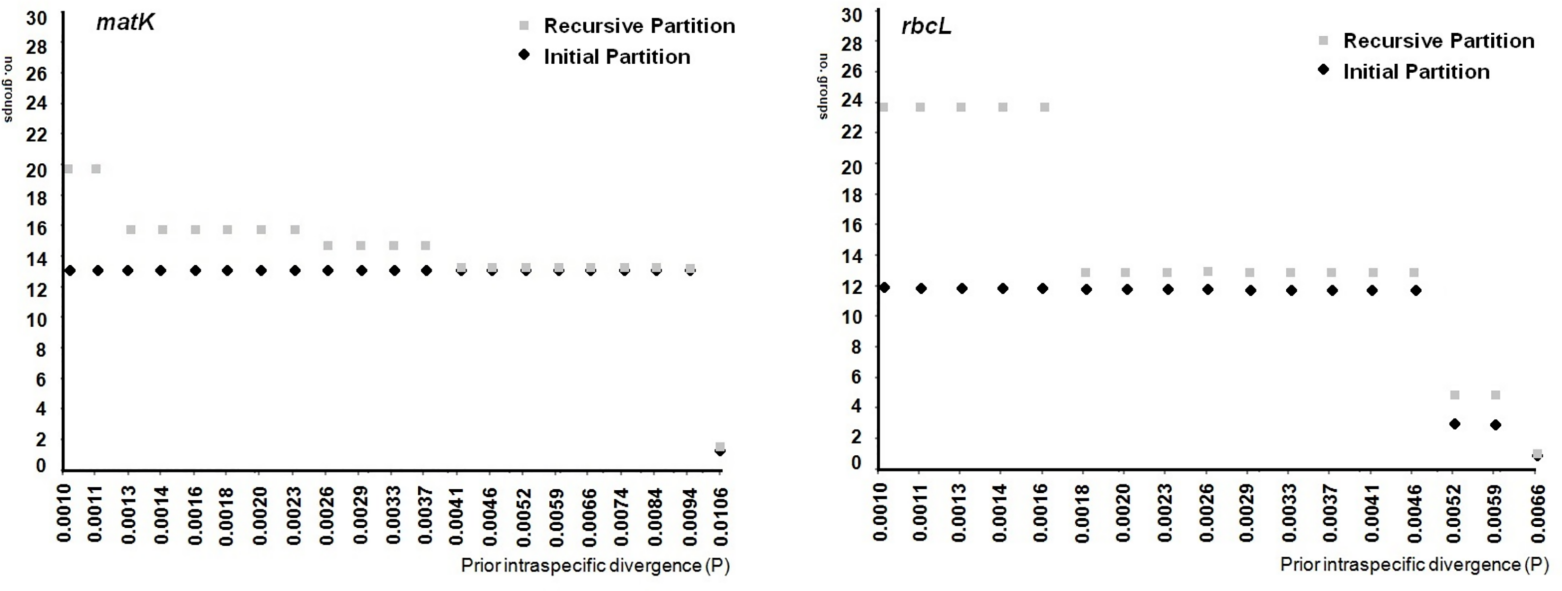
Automatic partition of the studied samples of *Aneura* spp. based on *matK* and *rbcL* loci. The number of groups, including *A*. *maxima* and *A*. *mirabilis*, resulted in initial and recursive partition at each given prior intraspecific divergence value were reported.

**Fig 6:**
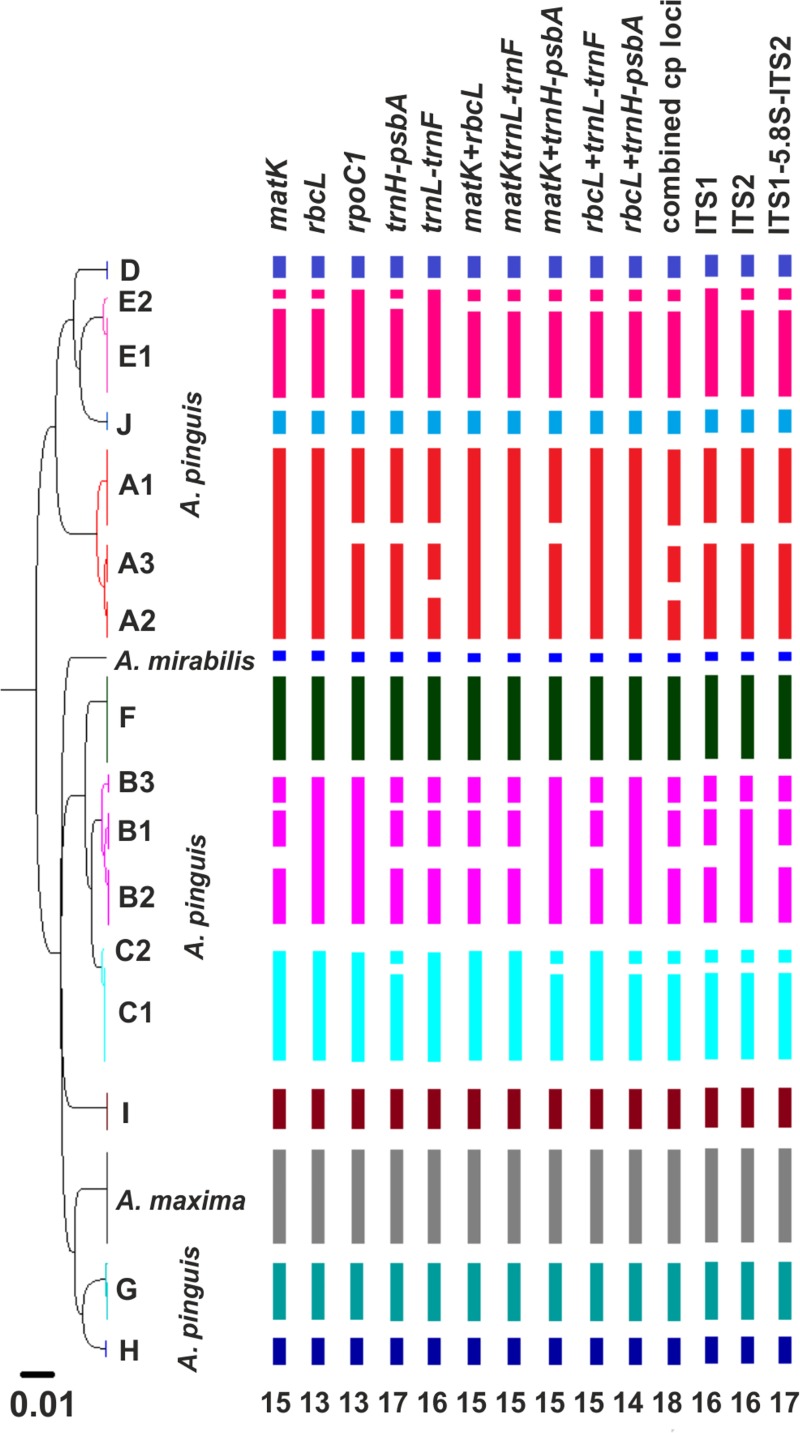
Ultrametric tree obtained by UPGMA analysis of the studied *Aneura* species generated from the combined dataset. The cryptic species of *A*. *pinguis* and the results of the ABGD analysis for the examined individual loci and their combinations were marked in different colors. The numbers below the diagram represent the number of groups detected as recursive partitions in ABGD.

In the ABGD analysis, six to 13 groups were identified within *A*. *pinguis* as initial partitions, depending on the locus. In the K2P model, the *rbcL* locus, two 2-gene combinations (*matK* + *trnH-psbA*, *rbcL* + *trnH-psbA*) and combined plastid loci produced one initial partition that contained always the same 10 groups of *A*. *pinguis* (plus one group of *A*. *maxima* and one of *A*. *mirabilis*) with intraspecific values in the range of 0.46% to 0.94% (Fig 5). The groups formed by the ABGD method were congruent with the groups created on the basis of phylogenetic trees, and they corresponded to the detected cryptic species A-J (Figs 1 and 6). All *A*. *pinguis* samples were assigned to the same group that was created on the basis of phylogenetic trees. The *matK* locus and the *rbcL* + *matK* combination produced 11 groups of *A*. *pinguis* corresponding to cryptic species A-J, and species B were split into two groups. The highest number of groups (13) was produced by *trnH-psbA* which split species A, B and C into two groups. The *trnL-trnF* locus, *matK* + *trnL-trnF* and *rbcL* + *trnL-trnF* combinations, and both nuclear regions (ITS1, ITS2) produced nine groups as the initial partition with P values of 0.59–2.15%. The combinations of *trnL-trnF*, *matK* + *trnL-trnF* and *rbcL* + *trnL-trnF* did not separate cryptic species B and C, whereas ITS1 and ITS2 did not distinguish species B and F. In the JC69 model, the results of the ABGD analysis were highly similar to those in the K2P model, except for *rpoC1*. In the K2P model, the *rpoC1* locus as the initial partition produced only 6 groups with P values of up to 1.06%, and it did not recognize species pairs B-F, E-J and G-H, whereas in the JC69 model, the *rpoC1* locus produced nine groups (P = 0.74%) and did not differentiate the species pair B-C. In all tested loci, *A*. *maxima* and *A*. *mirabilis* formed separate groups in the initial partition. In the ABGD analysis, data are first divided into groups as the initial partition based on a statistically inferred barcode gap, and the same procedure is then applied to the groups obtained in the first step to form a recursive partition. In all studied loci, recursive partitions resulted in 11 (*rbcL* and *rpoC1*) to 15 (*trnH-psbA*) groups of *A*. *pinguis* which split cryptic species A, B, C and E into three or two groups (Fig 6). However, only combined plastid loci distinguished between all groups in cryptic species A, B, C and E with P values from 0.17%. When the uncorrected p-distance was used, the ABGD analysis produced identical groups, but the P value of prior intraspecific differences was lower than that in K2P and JC69 models.

### Distribution of *A*. *pinguis* cryptic species

A comparison of the sequences obtained from the studied samples (Table 1) with GenBank sequences points to a wider distribution of individual cryptic species of *A*. *pinguis* in the world (S1 Fig). Plants belonging to cryptic species A occur also in the UK (A3), Portugal (A2 haplotypes with one and two substitutions) and New Zealand. Plants corresponding to cryptic species B were noted in the USA (B1), Costa Rica (B2), UK and Germany (B3). Cryptic species C and E were observed in Canada and Germany. Haplotypes identical to species F were found in the UK and USA (haplotypes with two and three substitutions in the USA). To date, cryptic species G, H and I have been found exclusively in Poland. Moreover, GenBank sequences harbored new haplotypes which formed separate clades, not identified in the samples examined in the present study. These haplotypes were found in North America (USA), Central America (Dominican Republic), South America (Ecuador), Asian Russia and Japan.

The cryptic species of *A*. *pinguis* clearly differ across various habitats (Table 2). The lineages of species A (A1, A2, A3) grows mainly on humus developed on limestone rocks, lineages of species B (B1, B2, B3) and F occur mainly on clay soils. The lineages C1 and C2 occupies mostly wet sandy soils, including on the shores of oligotrophic lakes, river and mountain stream banks and the lineages E1 and E2 thrives on calcareous rocks in flowing water. Species G, H and I are found in peat bogs.

## Discussion

### Identification of cryptic species of *A*. *pinguis* by DNA barcoding

DNA barcoding revealed that the nominally cosmopolitan *A*. *pinguis* was composed of 10 cryptic species, five of which had been previously described (signet A to E) [9, 41] and five were completely new (F to J). Furthermore, intraspecific differentiation was observed within four cryptic species A, B, C, and E. We identified 3 subgroups in cryptic species A and B (A1, A2, A3 and B1, B2, B3 respectively), and two subgroups in cryptic species C and E (C1, C2 and E1, E2, respectively). A total of 16 lineages in different evolutionary stages were distinguished within *A*. *pinguis*. In our study, groups A1, B1, C1and E1 corresponded to the previously described cryptic species A, B, C and E, respectively. Greater differentiation within *A*. *pinguis* can be explained by the fact that the analyzed material originated from a larger geographic area, and that the barcoding method delivers more accurate results than isozyme electrophoresis. Each of the tested loci in phylogenetic trees and network clusters show that the cryptic species of *A*. *pinguis* and *A*. *maxima* and *A*. *mirabilis* are a monophyletic clades (Figs 1 and 2; S1 and S2 Figs).

This study confirms the high potential of DNA barcoding for resolving taxonomic problems, and it demonstrates that DNA barcoding is a useful tool that complements the classical taxonomy of liverworts. We tested the core plant barcode (*rbcL* + *matK*) and five additional loci, including promising complementary barcodes (*trnH-psbA*, ITS and ITS2) in the cryptic species of *A*. *pinguis* and *A*. *maxima*. We also compared the sequences of the studied species with *A*. *mirabilis* sequences from GenBank [42]. The amplification efficiency of all sequences was 100%. High quality DNA was obtained for all (*matK*, *trnL-trnF*, ITS1, ITS2) or nearly all (*rbcL*, *rpoC1* and *trnH-psbA*) of the examined samples. All tested loci had 100% discriminant power to distinguish the studied species, they fulfilled the criteria of barcode DNA. None of the tested DNA regions alone had the power to detect all lineages. The combination of the *trnL-trnF* locus (the only locus that identified lineage A2) with *trnH-psbA* or ITS2 (loci that split species C and E) detected all lineages. This result was supported by the outcome of the ABGD analysis which automatically finds the distance where the barcode gap is located and splits the sequence alignment dataset into candidate species [36]. The units identified by ABGD correspond to the cryptic species and lineages of *A*. *pinguis* resolved by the NJ tree and to *A*. *maxima* and *A*. *mirabilis*. Among the examined loci, *trnH-psb*A, *trn*L*-trn*F, *mat*K and both ITS regions were characterized by the highest species resolution in the ABGD analysis, whereas *rbc*L and *rpoC1* were least effective. The ABGD analysis also revealed that *trn*L*-trn*F, which was tested with universal primers and produced high amplification and sequencing success, is also a promising candidate barcode for *Aneura* species. The *trnH-psb*A, *trn*L*-trn*F and ITS loci, together or combined with other sequences, are frequently used to resolve taxonomic problems (including cryptic species) in closely related liverworts [8, 26, 34, 43–45], and they are potentially the best DNA barcodes for this group of plants.

### Genetic differentiation of *A*. *pinguis*

Interspecies divergence ranged from 1.220% to 6.377% in combined cpDNA sequences, from 1.558% to 12.973% in ITS, and from 1.45% to 7.41% in the combined dataset (Table 5). Notably, most divergence exceeded the 3% threshold typically encountered between congeneric species pairs recognized by morphological features [46]. Recently divergence of 3% or 2% is proposed in different taxa as a threshold between species [6]. However the use of arbitrary distance thresholds in taxonomy has been debated. In some cases arbitrary distance thresholds can to suffer from varying rates of false-positive and false-negative error, depending on the data [47]. For example in close relatives species the distance thresholds are often smaller than Hebert’s proposal–they can be less than 1% [48,49]. In our study, divergence was below 3%, but higher than 1.22% in only six out of 45 pairs of cryptic species, whereas more than half of the distances in pairwise comparisons were higher than 5%. Moreover, the average divergence among the cryptic species of *A*. *pinguis* exceeded intraspecific divergences 15-fold (Table 7). Hebert *et al*. [35] proposed the 10-fold rule as the standard sequence threshold, where the mean of interspecific distances should be more than 10-fold higher than the mean of intraspecific distances for the examined group. Our results point to clear genetic differences between the cryptic species of *A*. *pinguis*.

Phylogenetic analyses (Fig 1, S1 and S2 Figs) of the combined dataset consistently revealed that all cryptic species of *A*. *pinguis* as well as *A*. *maxima* and *A*. *mirabilis* (two taxonomically recognized species of *Aneura* genus) formed separate clades and that *A*. *maxima* and *A*. *mirabilis* were nested between different cryptic species of *A*. *pinguis*. These results correspond with previous molecular findings which demonstrated that *A*. *pinguis* is a paraphyletic taxon [17, 50–52]. In our study, the phylogenetic tree of *Aneura* was divided into two distinct clades. The first clade contained 6 cryptic species of *A*. *pinguis* (B, F, C, H, G and I) as well as *A*. *maxima* and *A*. *mirabilis*, whereas the second clade contained four cryptic species (A, D, E, J) of *A*. *pinguis*. The above suggests that the cryptic species of *A*. *pinguis* are not directly derived from one common ancestor and that their evolutionary history is more complex. Moreover, these two distinct evolutionary lines of *A*. *pinguis* had diverged before *A*. *maxima* and *A*. *mirabilis* were split. The division of *A*. *pinguis* into two major clades confirmed the results of the network analysis (Fig 2), where the two groups of cryptic species were separated from each other by at least 164 mutation steps. The analysis of K2P distances confirmed this thesis. In all analyzed DNA regions, the distances between most pairs of cryptic species of *A*. *pinguis* were greater than between *A*. *maxima* and *A*. *mirabilis* (Table 5). Wickett & Goffinet [50] postulated that *A*. *pinguis*, *A*. *maxima* and *A*. *mirabilis* could be regarded as a species complex. Indeed, this group appears to have a more complex taxonomy because *A*. *pinguis* is a complex of cryptic species and, as indicated by other authors [50,51], *A*. *maxima* is not a homogeneous taxon either.

### Geographic distribution, habitat preferences and morphological diversity

A comparison of the obtained sequences (*rbcL*, *trnL-trnF* and ITS) with *A*. *pinguis* sequences from GenBank indicates that in addition to the identified haplotypes, the analyzed sequences harbored other haplotypes which could suggest the presence of additional cryptic species of *A*. *pinguis* (S2 Fig). In this study, the distribution of *A*. *pinguis* was analyzed only within a limited range, therefore other cryptic species of *A*. *pinguis* could exist. New haplotypes forming separate clades were found in the USA, Dominican Republic, Ecuador, Asian Russia and Japan. To date, five (A, D, G, H, I) cryptic species have been found exclusively in Europe, of which three have been identified only in Poland (G, H and I). Species B, which grows in Europe, North, Central and South America, was the most sampled (most sequences were found in GenBank) and widespread species.

*A*. *pinguis* species differ not only in their geographic distribution, but also in habitat preferences. Minor differences between subgroups within cryptic species were found (Table 2). The cryptic species growing in peat bogs (G, H and I) were most highly correlated with habitat type. The lineage C1 was most tolerant and occupy the most different substrata. In our opinion, diversification within *A*. *pinguis* is clearly linked to individual species ecology, and it is indicative of stabilizing selection in different habitats. Moreover, the haplotypes in the ITS region indicate that cryptic species form reproductively isolated populations, even if they are largely sympatric, such as species A, B and C. A lack of recombinants in the cryptic species of *A*. *pinguis* also revealed a previous enzymatic study [9].

Similarly to earlier studies of cryptic species A, B and C [19], we struggled to find morphological features that would identify the remaining cryptic species of *A*. *pinguis*. Unfortunately, a biometric analysis of thalli in *A*. *pinguis* cryptic species did not reveal significant qualitative morphological differences between these cryptic species. We were only able to identify minor phenotypic diversity in morphology, especially in the size of the thallus. For example, species A, B and C were larger, whereas species E, H, G, I were rather smaller. The range of variation in thallus size is high, with partial overlap between the species. Therefore, this feature cannot be the basis for the identification of *A*. *pinguis* species, and it can only be used as a supportive characteristics. This observation is consistent with the findings of Schuster [23, 53] who stated that morphological varieties within *A*. *pinguis* are “virtually inseparable”. However some sporophyte characteristics, such as: seta anatomy, capsule wall structure and thickening pattern, spores, spore wall anatomy, elater features and spermatid architecture are less variable then gametophyte characters and therefore more valuable as taxonomic markers [54–56]. Thus, sporophyte features may be helpful for delimitation at the species level within the *A*. *pinguis* species complex.

The DNA barcode of *A*. *pinguis* reveals new cryptic species. They are impossible to distinguish using morphological methods alone. Bryophytes such as *A*. *pinguis* are structurally simple plants with a limited number of morphological traits, and they frequently include morphologically indistinguishable entities. From the point of view of traditional taxonomy, cryptic species cannot be classified as classical taxonomic species because they do not have unique morphological traits that correspond to genetic differentiation; however, they conform to the species concept due to a lack of recombination [4]. The accelerated rate of cryptic species detection in DNA sequencing suggests that molecular data should be incorporated into alpha taxonomy whenever possible. Integrative taxonomy which relies on collaborative and mutually beneficial integrative applications of molecular biology, such as DNA barcoding, comparative morphology and descriptive taxonomy, is recommended for describing species [7, 57–58]. According to some authors, ecological preferences and geographical distribution should also be taken into account in the newly detected molecular species [1, 36, 59, 60]. Most of the distinguished cryptic species of *A*. *pinguis* differed in their habitat preferences and geographical distribution, which appears to be an important consideration and provides additional evidence for the presence of a new biological species in the genus *Aneura*.

## Supporting information

S1 Table Collection details, GenBank accession numbers of the *Aneura* samples used in the DNA barcode studies. *Samples from herbarium collection, ^a-f^ references for sequences from GenBank, N–number of sequences obtained in present studies.
(DOC)

S2 Table Sequences of primers used in the present study. (DOC)

S3 Table Average genetic divergences (K2P %) for *A*. *pinguis* cryptic species, *A*. *maxima* and *A*. *mirabilis*; combined plastid sequences (below diagonal) and ITS (a bove diagonal). (DOC)

S4 Table Average genetic divergences (K2P %) for *A*. *pinguis* lineages, *A*. *maxima* and *A*. *mirabilis*; combined plastid sequences (below diagonal) and ITS (above diagonal). (DOC)

S1 Fig Neighbor-joining 75% majority-rule bootstrap consensus trees for the studied species of *Aneura* genus. (PDF)

S2 Fig **Neighbor joining (A) and maximum parsimony (B) consensus trees of *Aneura*. *pinguis* cryptic species based on a combined dataset.** *Aneura maxima* and *A*. *mirabilis* were used for comparison. *Pellia endiviifolia* was used as an outgroup. Only the accessions with the sequences obtained for all loci were included in the analysis. Bootstrap values above 85% are indicated above branches.
(PDF)

S3 Fig Intraspecific and interspecific pairwise K2P distances for individual loci and their combinations for *Aneura pinguis*. (PDF)
