# Supplementary material for: DNA barcoding, ecology and geography of the cryptic species of Aneura pinguis and their relationships with Aneura maxima and Aneura mirabilis (Metzgeriales, Marchantiophyta)
Source: PLoS One. 2017 Dec 5;12(12):e0188837. doi: 10.1371/journal.pone.0188837 (PMC5716573; doi:10.1371/journal.pone.0188837)
Supplement: S1 Table — *Samples from herbarium collection, a-f references for sequences from GenBank, N–number of sequences obtained in present studies. (DOC) [file pone.0188837.s001.doc]

**S1 Table** Plant material, collection details and GenBank accession numbers of the material used in the analysis of DNA barcodes in *Aneura*. *Samples from herbarium collection, ^a-f^ references for sequences from GenBank, N – number of sequences obtained in present studies.

| **ID No.** | **Voucher number** | **Geographic coordinates** | **Geographic region** | ***matK* N=118** | ***rbcL* N=102** | ***rpoC1***  **N=70** | ***psbA-trnH***  **N=90** | ***trnL-trnF***  **N=144** | **ITS1-5.8S-ITS2 N=119** |  | | |
| --- | --- | --- | --- | --- | --- | --- | --- | --- | --- | --- | --- | --- |
| ***Aneura pinguis*** | | | | | | | | | |  | | |
| A1-1 | POZW 42860 | 49°06’N  22°41’E | SE Poland, Bieszczady Mts, valley of Terebowiec stream | KY705529 | KY705737 | − | − | KY705909 | KY705410 |  | | |
| A1-2 | POZW 42798 | 49°06’N  22°41’E | SE Poland, Bieszczady Mts, valley of Terebowiec stream | KY705530 | KY705738 | KY705839 | KY705647 | KY705910 | KY705411 |  | | |
| A1-3 | POZW 42485 | 49°27’N  20°27’E | S Poland, Beskidy Mts, Szczawa stream | KY705531 | KY705739 | − | KY705648 | KY705911 | KY705412 |  | | |
| A1-4 | POZW 42486 | 49°27’N  20°27’E | S Poland, Beskidy Mts, Szczawa stream | KY705532 | KY705740 | KY705840 | KY705649 | KY705912 | KY705413 |  | | |
| A1-5 | POZW 42903 | 49°26’N  20°26’E | S Poland, Beskidy Mts, valley of Kozłecki stream | KY705533 | KY705741 | − | − | KY705913 | KY705414 |  | | |
| A1-6* | POZW 42487 | 49°27’N  20°27’E | S Poland, Beskidy Mts, Szczawa stream | − | − | − | − | KY705914 | − |  | | |
| A1-7 | POZW 42861 | 49°25’N  20° 21’E | S Poland, Pieniny Mts, Limbargowy Potok stream | KY705534 | KY705742 | − | KY705650 | KY705915 | KY705415 |  | | |
| A1-8 | POZW 42904 | 49°25’N  20° 20’E | S Poland, Pieniny Mts, Barbarzyna meadow | KY705535 | KY705743 | − | − | KY705916 | KY705416 |  | | |
| A1-9 | POZW 40340 | 49°16’N  19° 49’E | S Poland, Tatry Mts, Wielka Sucha Dolina valley | KY705536 | KY705744 | − | KY705651 | KY705917 | KY705417 |  | | |
| A1-10 | POZW 42907 | 49°16’N  19°59’E | S Poland, Tatry Mts, Olczyska Valley | KY705537 | KY705745 | KY705841 | KY705652 | KY705918 | KY705418 |  | | |
| A1-11 | POZW 42862 | 49°15’N  20°00’E | S Poland, Tatry Mts, NE slope of Skupniów Upłaz Mt. | KY705538 | KY705746 | − | KY705653 | KY705919 | KY705419 |  | | |
| A2-1 | POZW 42478 | 49°26’N  20°26’E | S Poland, Beskidy Mts, valley of Kozłecki stream | KY705539 | KY705747 | KY705842 | KY705654 | KY705920 | KY705420 |  | | |
| A2-2 | POZW 42863 | 49°26’N  20°26’E | S Poland, Beskidy Mts, Wygon stream | KY705540 | KY705748 | KY705843 | KY705655 | KY705921 | KY705421 |  | | |
| A2-3* | POZW 42502 | 49°27’N  20°26’E | S Poland, Beskidy Mts, Wygon stream | − | − | − | − | KY705922 | − |  | | |
| A2-4 | POZW 42905 | 49°25’N  20°2 4’E | S Poland, Pieniny Mts, Pieniński Potok stream | KY705541 | KY705749 | KY705844 | KY705656 | KY705923 | KY705422 |  | | |
| A2-5 | POZW 42864 | 49°16’N  19°59’E | S Poland, Tatry Mts, Olczyska Valley | KY705542 | − | − | KY705657 | KY705924 | KY705423 |  | | |
| A3-1 | POZW 40383 | 49°24’N  20°33’E | S Poland, Pieniny Mts, tributary of Potok Skalskie stream | KY705543 | KY705750 | KY705845 | KY705658 | KY705925 | KY705424 |  | | |
| A3-2 | POZW 42133 | 49°24’N  20°33’E | S Poland, Pieniny Mts, tributary of Potok Skalskie stream | KY705544 | KY705751 | KY705846 | KY705659 | KY705926 | KY705425 |  | | |
| A3-3 | POZW 40331 | 49°15’N  19°59’E | S Poland, Tatry Mts, NE slope of Skupniów Upłaz Mt. | KY705545 | KY705752 | − | KY705660 | KY705927 | KY705426 |  | | |
| A3-4 | POZW 41048 | 49°15’N  19°59’E | S Poland, Tatry Mts, NE slope of Skupniów Upłaz Mt. | KY705546 | KY705753 | KY705847 | KY705661 | KY705928 | KY705427 |  | | |
| A3-5 | POZW 42822 | 49°15’N  20°02’E | S Poland, Tatry Mts, Pańszczyca valley | KY705547 | KY705754 | − | − | KY705929 | KY705428 |  | | |
| A3-6 | POZW 42865 | 49°15’N  20°02’E | S Poland, Tatry Mts, Pańszczyca valley | KY705548 | KY705755 | − | − | KY705930 | KY705429 |  | | |
| A3-7 | POZW 42866 | 49°16’N  20°01’E | S Poland, Tatry Mts, Sucha Woda Valley | KY705549 | KY705756 | − | − | KY705931 | KY705430 |  | | |
| A3-8 | POZW 42821 | 49°16’N  19°57’E | S Poland, Tatry Mts, valley of Biały Potok stream | KY705550 | KY705757 | KY705848 | KY705662 | KY705932 | KY705431 |  | | |
| A3-9 | POZW 42906 | 49°15’N  19°59’E | S Poland, Tatry Mts, NE slope of Skupniów Upłaz Mt. | KY705551 | KY705758 | KY705849 | KY705663 | KY705933 | KY705432 |  | | |
| A3-10 | POZW 42867 | 49°15’N  19°59’E | S Poland, Tatry Mts, NE slope of Skupniów Upłaz Mt. | KY705552 | KY705759 | − | − | KY705934 | KY705433 |  | | |
| A3-11 | POZW 42868 | 49°15’N  19°59’E | S Poland, Tatry Mts, NE slope of Skupniów Upłaz Mt. | KY705553 | KY705760 | − | − | KY705935 | KY705434 |  | | |
| A3-12 | POZW 42869 | 49°16’N  19°56’E | S Poland, Tatry Mts, Strążyska Valley | KY705554 | KY705761 | − | KY705664 | KY705936 | KY705435 |  | | |
| A3-13* | POZW 40438 | 49°15’N  20°00’E | S Poland, Tatry Mts, NE slope of Skupniów Upłaz Mt. | − | − | − | − | KY705937 | − |  | | |
| A3-14* | POZW 40435 | 49°15’N  20°00’E | S Poland, Tatry Mts, NE slope of Skupniów Upłaz Mt. | − | − | − | − | KY705938 | − |  | | |
| A3-15* | POZW 40097 | 49°15’N  19°59’E | S Poland, Tatry Mts, NE slope of Skupniów Upłaz Mt. | − | − | − | − | KY705939 | − |  | | |
| A3-16* | POZW 25700 | 49°25’N  19°59’E | S Poland, Tatry Mts, Jaworzynka valley | − | − | − | − | KY705940 | − |  | | |
| A3-17* | POZW 25707 | 49°14’N  19°52’E | S Poland, Tatry Mts, Kościeliska valley | − | − | − | − | KY705941 | − |  | | |
| A3-18* | CHR 542474 | no data available | New Zealand, Wairarapa, Ruakokopatuna, coll. D. Glenny | − | − | − | − | KY705942 | − |  | | |
| A3^a^ |  |  | United Kingdom | − | − | − | − | − | DQ986144 |  | | |
| A3^b^ |  |  | United Kingdom | − | EF547816 | − | − | − | − |  | | |
| A^c^ |  |  | Portugal | − | − | − | − | FM210486 | − |  | | |
| A^c^ |  |  | Portugal | − | − | − | − | FM210487 | − |  | | |
| B1-1 | POZW 40261 | 58°48’N 20°24’E | NE Poland, Warmia, Lake Redykajny | KY705555 | KY705762 | KY705850 | KY705665 | KY705943 | KY705436 |  | | |
| B1-2 | POZW 42408 | 54°16’N  17°30’E | NW Poland, Western Pomerania, Lake Witno | KY705556 | KY705763 | KY705851 | KY705666 | KY705944 | KY705437 |  | | |
| B1-3 | POZW 42019 | 52°22’N  16° 51’E | Central Poland, Wielkopolska, SW part of Poznań | KY705557 | KY705764 | KY705852 | KY705667 | KY705945 | KY705438 |  | | |
| B1-4 | POZW 42791 | 49°06’N  22°31’E | SE Poland, Bieszczady Mts, valley of Górna Solinka river | KY705558 | KY705765 | KY705853 | KY705668 | KY705946 | KY705439 |  | | |
| B1-5 | POZW 42870 | 49°06’N  22°31’E | SE Poland, Bieszczady Mts, valley of Górna Solinka stream | KY705559 | KY705766 | − | − | KY705947 | KY705440 |  | | |
| B1^b^ |  |  | USA | − | EF547813 | − | − | − | − |  | | |
| B2-1 | POZW 42793 | 49°06’N  22°29’E | SE Poland, Bieszczady Mts, valley of Beskidnik stream | KY705560 | KY705767 | KY705854 | KY705669 | KY705948 | KY705441 |  | | |
| B2-2 | POZW 42795 | 49°06’N  22°29’E | SE Poland, Bieszczady Mts, valley of Beskidnik stream | KY705561 | KY705768 | KY705855 | KY705670 | KY705949 | KY705442 |  | | |
| B2-3* | POZW 40210 | 49°06’N  22°31’E | SE Poland, Bieszczady Mts, valley of Górna Solinka stream | − | − | − | − | KY705950 | − |  | | |
| B2-4* | POZW 40214 | 49°06’N  22°41’E | SE Poland, Bieszczady Mts, valley of Górna Solinka stream | − | − | − | − | KY705951 | − |  | | |
| B2-5 | POZW 40341 | 49°16’N  19°49’E | S Poland, Tatry Mts, Wielka Sucha Dolina valley | KY705562 | KY705769 | KY705856 | KY705671 | KY705952 | KY705443 |  | | |
| B2-6 | POZW 40538 | 49°16’N  19°49’E | S Poland, Tatry Mts, Wielka Sucha Dolina valley | KY705563 | KY705770 | KY705857 | KY705672 | KY705953 | KY705444 |  | | |
| B2-7* | POZW 29474 | 49°17’N  20°02’E | S Poland, Las Capowski forest at the foot of Tatry Mts | − | − | − | − | KY705954 | − |  | | |
| B2^b^ |  |  | Costa Rica | − | EF547821 | − | − | − | − |  | | |
| B3-1 | POZW 41412 | 54°05’N  17°57’E | NW Poland, Western Pomerania, Lake Książe | KY705564 | KY705771 | KY705858 | KY705673 | KY705955 | KY705445 |  | | |
| B3-2 | POZW 42765 | 54°05’N  17°57’E | NW Poland, Western Pomerania, Lake Książe | KY705565 | KY705772 | KY705859 | KY705674 | KY705956 | KY705446 |  | | |
| B3-3 | POZW 42769 | 54°05’N  17°57’E | NW Poland, Western Pomerania, Lake Książe | KY705566 | KY705773 | − | KY705675 | KY705957 | KY705447 |  | | |
| B3-4 | POZW 42762 | 54°15’N  17°29’E | NW Poland, Western Pomerania, Słupia river | KY705567 | KY705774 | − | KY705676 | KY705958 | KY705448 |  | | |
| B3-5 | POZW 42911 | 54°15’N  17°29’E | NW Poland, Western Pomerania, Słupia river | KY705568 | KY705775 | KY705860 | KY705677 | KY705959 | KY705449 |  | | |
| B3^a^ |  |  | United Kingdom | − | − | − | − | − | DQ986141 |  | | |
| B3^b^ |  |  | United Kingdom | − | EF547813 | − | − | − | − |  | | |
| B3^c^ |  |  | Germany | − | − | − | − | FM210489 | − |  | | |
| B^d^ |  |  | Russia | − | DQ439675 | − | − | − | − |  | | |
| C1-1 | POZW 42872 | 54°07’N  17°53’E | NW Poland, Western Pomerania, Lake Garczyn | KY705569 | − | KY705861 | KY705678 | KY705960 | KY705450 |  | | |
| C1-2 | POZW 42763 | 54°07’N  17°53’E | NW Poland, Western Pomerania, Lake Garczyn | KY705570 | − | − | − | KY705961 | KY705451 |  | | |
| C1-3 | POZW 42873 | 54°07’N  17°54’E | NW Poland, Western Pomerania, Lake Garczyn | KY705571 | − | − | KY705679 | KY705962 | KY705452 |  | | |
| C1-4 | POZW 42761 | 54°15’N  17°29’E | NW Poland, Western Pomerania, Słupia river | KY705572 | − | − | − | KY705963 | KY705453 |  | | |
| C1-5 | POZW 42874 | 54°15’N  17°29’E | NW Poland, Western Pomerania, Słupia river | KY705573 | KY705776 | − | − | KY705964 | KY705454 |  | | |
| C1-6 | POZW 42912 | 54°04’N  17°51’E | NW Poland, Western Pomerania, Lake Kulkówko | KY705574 | KY705777 | KY705862 | KY705680 | KY705965 | KY705455 |  | | |
| C1-7 | POZW 42747 | 53°23’N  16°35’E | Central Poland, Wielkopolska, valley of Rurzyca river | KY705575 | KY705778 | KY705863 | KY705681 | KY705966 | KY705456 |  | | |
| C1-8 | POZW 42749 | 53°23’N  16°35’E | Central Poland, Wielkopolska, valley of Rurzyca river | KY705576 | KY705779 | KY705864 | KY705682 | KY705967 | KY705457 |  | | |
| C1-9 | POZW 42875 | 49°07’N  22°29’E | SE Poland, Bieszczady Mts, banks of Górna Solinka stream | KY705577 | KY705780 | KY705865 | KY705683 | KY705968 | KY705458 |  | | |
| C1-10 | POZW 40539 | 49°16’N  20°01’E | S Poland, Tatry Mts, Sucha Woda Valley | KY705578 | KY705781 | KY705866 | KY705684 | KY705969 | KY705459 |  | | |
| C1-11 | POZW 42819 | 49°16’N  20°01’E | S Poland, Tatry Mts, Sucha Woda Valley | KY705579 | KY705782 | − | − | KY705970 | KY705460 |  | | |
| C1-12 | POZW 42876 | 49°15’N  20°00’E | S Poland, Tatry Mts, NE slope of Skupniów Upłaz Mt. | KY705580 | KY705783 | − | − | KY705971 | KY705461 |  | | |
| C1-13 | POZW 42908 | 49°15’N  20°02’E | S Poland, Tatry Mts, Pańszczyca Valley | KY705581 | KY705784 | KY705867 | KY705685 | KY705972 | KY705462 |  | | |
| C1-14 | POZW 42877 | 49°16’N  19°57’E | S Poland, Tatry Mts, valley of Biały Potok stream | KY705582 | − | − | − | KY705973 | KY705463 |  | | |
| C1-15 | POZW 42878 | 49°15’N  20°00’E | S Poland, Tatry Mts, NE slope of Skupniów Upłaz Mt. | KY705583 | − | KY705868 | − | KY705974 | KY705464 |  | | |
| C2-1 | POZW 42792 | 49°06’N  22°31’E | SE Poland, Bieszczady Mts, valley of Górna Solinka stream | KY705584 | KY705785 | KY705869 | KY705686 | KY705975 | KY705465 |  | | |
| C2-2 | POZW 42879 | 49°05’N  22°38’E | SE Poland, Bieszczady Mts, Ustrzyki Górne, small stream | KY705585 | KY705786 | KY705870 | KY705687 | KY705976 | KY705466 |  | | |
| C2-3 | POZW 42880 | 49°05’N  22°38’E | SE Poland, Bieszczady Mts, Ustrzyki Górne, small stream | KY705586 | KY705787 | − | KY705688 | KY705977 | KY705467 |  | | |
| C^b^ |  |  | Canada | − | EF547817 | − | − | − | − |  | | |
| D 1 | POZW 40163 | 55°16’N  5°29’E | United Kingdom, Scotland, North Ebudes, coll. D.G. Long | KY705587 | KY705788 | KY705871 | KY705689 | KY705978 | KY705468 |  | | |
| D 2 | POZW 40238 | 55°34’N  9°03’E | United Kingdom, Ireland, West Galway, coll. D.G. Long | KY705588 | KY705789 | KY705872 | KY705690 | KY705979 | KY705469 |  | | |
| E1-1 | POZW 41031 | 49°16’N  19°57’E | S Poland, Tatry Mts, valley of Biały Potok stream | KY705589 | − | − | KY705691 | KY705980 | KY705470 |  | | |
| E1-2 | POZW 40521 | 49°16’N  19°49’E | S Poland, Tatry Mts, Wielka Sucha Dolina valley | KY705590 | KY705790 | KY705873 | KY705692 | KY705981 | KY705471 |  | | |
| E1-3 | POZW 40520 | 49°15’N  20°02’E | S Poland, Tatry Mts, Pańszczyca Valley | KY705591 | − | − | KY705693 | KY705982 | KY705472 |  | | |
| E1-4 | POZW 42439 | 49°16’N  19°57’E | S Poland, Tatry Mts, valley of Biały Potok stream | KY705592 | − | − | KY705694 | KY705983 | KY705473 |  | | |
| E1-5 | POZW 42440 | 49°16’N  19°57’E | S Poland, Tatry Mts, valley of Biały Potok stream | KY705593 | − | − | KY705695 | KY705984 | KY705474 |  | | |
| E1-6 | POZW 42881 | 49°16’N  19°49’E | S Poland, Tatry Mts, Wielka Sucha Dolina valley | KY705594 | KY705791 | − | − | KY705985 | KY705475 |  | | |
| E1-7 | POZW 42824 | 49°16’N  19°49’E | S Poland, Tatry Mts, Wielka Sucha Dolina valley | KY705595 | KY705792 | KY705874 | KY705696 | KY705986 | KY705476 |  | | |
| E1-8 | POZW 40507 | 49°16’N  19°57’E | S Poland, Tatry Mts, valley of Biały Potok stream | KY705596 | − | − | KY705697 | KY705987 | KY705477 |  | | |
| E1-9 | POZW 42882 | 49°16’N  19°57’E | S Poland, Tatry Mts, valley of Biały Potok stream | KY705597 | KY705793 | − | − | KY705988 | KY705478 |  | | |
| E1-10 | POZW 42883 | 49°16’N  19°57’E | S Poland, Tatry Mts, valley of Biały Potok stream | KY705598 | KY705794 | − | − | KY705989 | KY705479 |  | | |
| E1-11 | POZW 42884 | 49°16’N  19°57’E | S Poland, Tatry Mts, valley of Biały Potok stream | KY705599 | KY705795 | − | − | KY705990 | KY705480 |  | | |
| E1-12 | POZW 42909 | 49°16’N  19°57’E | S Poland, Tatry Mts, valley of Biały Potok stream | KY705600 | KY705796 | KY705875 | KY705698 | KY705991 | KY705481 |  | | |
| E1-13 | POZW 42885 | 49°16’N  19°57’E | S Poland, Tatry Mts, valley of Biały Potok stream | KY705601 | KY705797 | KY705876 | KY705699 | KY705992 | KY705482 |  | | |
| E1-14 | POZW 42886 | 49°16’N  19°57’E | S Poland, Tatry Mts, valley of Biały Potok stream | KY705602 | KY705798 | − | − | KY705993 | KY705483 |  | | |
| E1-15* | POZW 16017 | 49°16’N  19°49’E | S Poland, Tatry Mts, Wielka Sucha Dolina valley | − | − | − | − | KY705994 | − |  | | |
| E1-16* | POZW 15992 | 49°15’N  20°02’E | S Poland, Tatry Mts, Pańszczyca Valley | − | − | − | − | KY705995 | − |  | | |
| E1-17* | POZW 25552 | 49°15’N  20°02’E | S Poland, Tatry Mts, Pańszczyca Valley | − | − | − | − | KY705996 | − |  | | |
| E1-18 | POZW 42887 | 50°14’N  19°01’E | S Poland, Śląsk, near Katowice | KY705603 | KY705799 | KY705877 | KY705700 | KY705997 | KY705484 |  | | |
| E1-19* | B 191401 | 69°11’N  104°42’W | Canada, Mt. Pelly, Cambridge Bay area Victoria Island, coll. F.M. Boas | − | − | − | − | KY705998 | − |  | | |
| E1-20* | B 203267 | 59°44’N  133°46’W | Canada, British Columbia, Atlin Lake, coll. P. Williston | − | − | − | − | KY705999 | − |  | | |
| E1-21* | POZW 15980 | 53°18’N  14°40’E | NW Poland, Western Pomerania, beech forest near Szczecin | − | − | − | − | KY706000 | − |  |  |  |
| E2-1 | POZW 42888 | 49°25’N  20° 21’E | S Poland, Pieniny Mts, Limbargowy Potok stream | KY705604 | KY705800 | KY705878 | KY705701 | KY706001 | − |  |  |  |
| E^c^ |  |  | Germany | − | − | − | − | FM210485 | − |  | | |
| F 1 | POZW 42889 | 53°57’N  14°29’E | NW Poland, Wolin Island, Gosań Mt, on a cliff | KY705605 | KY705801 | KY705879 | KY705702 | KY706002 | KY705486 |  | | |
| F 2 | POZW 42890 | 53°57’N  14°30’E | NW Poland, Wolin Island, Świdna Kępa Mt, on a cliff | KY705606 | − | − | − | KY706003 | KY705487 |  | | |
| F 3* | POZW 16086 | 53°57’N  14°30’E | NW Poland, Wolin Island, Gosań Mt, on a cliff | − | − | − | − | KY706004 | − |  | | |
| F 4* | POZW 40182 | 54°06’N  23°05’E | NE Poland, Suwałki Lake District, Lake Pierty | − | − | − | − | KY706005 | − |  | | |
| F 5 | POZW 42901 | 49°09’N  22°34’E | SE Poland, Bieszczady Mts, Old quarry near Brzegi Górne | KY705607 | KY705802 | KY705881 | KY705704 | KY706006 | KY705488 |  | | |
| F 6 | POZW 42771 | 49°09’N  22°34’E | SE Poland, Bieszczady Mts, Old quarry near Brzegi Górne | KY705608 | KY705803 | KY705882 | KY705705 | KY706007 | KY705489 |  | | |
| F 7 | POZW 42797 | 49°06’N  22°41’E | SE Poland, Bieszczady Mts, valley of Terebowiec stream | KY705609 | KY705804 | − | − | KY706008 | KY705490 |  | | |
| F 8 | POZW 42891 | 49°07’N  22°40’E | SE Poland, Bieszczady Mts, tributary of Wołosaty stream | KY705610 | KY705805 | KY705883 | KY705706 | KY706009 | KY705491 |  | | |
| F 9 | POZW 42892 | 49°07’N  22°40’E | SE Poland, Bieszczady Mts, tributary of Wołosaty stream | KY705611 | KY705806 | − | − | KY706010 | KY705492 |  | | |
| F 10 | POZW 42893 | 49°07’N  22°40’E | SE Poland, Bieszczady Mts, tributary of Wołosaty stream | KY705612 | KY705807 | − | − | KY706011 | KY705493 |  | | |
| F 11 | POZW 42495 | 49°26’N  20°26’E | S Poland, Beskidy Mts, valley of Kozłecki stream | KY705613 | KY705808 | KY705884 | KY705707 | KY706012 | KY705494 |  | | |
| F 12 | POZW 42818 | 49°26’N  20°26’E | S Poland, Beskidy Mts, valley of Kozłecki stream | KY705614 | KY705809 | KY705885 | KY705708 | KY706013 | KY705495 |  | | |
| F 13 | POZW 42894 | 49°26’N  20°26’E | S Poland, Beskidy Mts, valley of Kozłecki stream | KY705615 | KY705810 | KY705886 | KY705709 | KY706014 | KY705496 |  | | |
| F 14 | POZW 42910 | 49°24’N  20° 24’E | S Poland, Pieniny Mts, Kotłowy Potok stream | KY705616 | KY705811 | KY705887 | KY705710 | KY706015 | KY705497 |  | | |
| F 15 | POZW 42895 | 49°16’N  19°57’E | S Poland, Tatry Mts, valley of Biały Potok stream | KY705617 | − | KY705888 | − | KY706016 | KY705498 |  | | |
| F 16 | POZW 16062 | 50°14’N  16°57’E | SW Poland, Góry Bialskie Mts | − | − | − | − | KY706017 | − |  | | |
| F 17 | NYGB 263899 | 45°32’N  84°40’W | USA, Michigan, Cheboygan Co., N of Horgsback Road, coll. W.R. Buck | − | − | − | − | KY706018 | − |  | | |
| F 18 | NYGB 576889 | 45°29’N  83°58’W | USA, Presque Isle Country, Lake Huron, N of Evergreen Highway, coll. W.R. Buck | − | − | − | − | − | KY705499 |  | | |
| F^a^ |  |  | USA | − | − | − | − | − | DQ986142 |  | | |
| F^b^ |  |  | United Kingdom | − | EF547822 | − | − | − | − |  | | |
| F^b^ |  |  | USA | − | EF547815 | − | − | − | − |  | | |
| G 1* | POZW 30982 | 54°06’N  23°21’E | NE Poland, Suwałki Lake District, lake Druce | − | − | − | − | KY706019 | − |  | | |
| G 2* | POZW 30983 | 54°06’N  23°21’E | NE Poland, Suwałki Lake District, lake Druce | − | − | − | − | KY706020 | − |  | | |
| G 3 | POZW 42240 | 54°16’N  17°29’E | NW Poland, Western Pomerania, Lake Duże Witno | KY705618 | KY705812 | KY705889 | KY705711 | KY706021 | KY705500 |  | | |
| G 4 | POZW 42241 | 54°16’N  17°29’E | NW Poland, Western Pomerania, Lake Duże Witno | KY705619 | KY705813 | − | − | KY706022 | KY705501 |  | | |
| G 5 | POZW 42767 | 54°05’N  17°51’E | NW Poland, Western Pomerania, Lake Małe Płocice | KY705620 | KY705814 | KY705890 | KY705712 | KY706023 | KY705502 |  | | |
| G 6 | POZW 42896 | 49°03’N  22°46’E | SE Poland, Bieszczady Mts, Beskid Pass | KY705621 | KY705815 | KY705891 | KY705713 | KY706024 | KY705503 |  | | |
| G 7 | POZW 42897 | 49°36’N  19°31’E | S Poland, Beskidy Mts, Dejakowy Potok stream | KY705622 | KY705816 | KY705892 | KY705714 | KY706025 | KY705504 |  | | |
| H 1* | POZW 40187 | 54°07’N  23°19’E | NE Poland, Suwałki Lake District, Lake Sejny | − | − | − | − | KY706026 | − |  | | |
| H 2* | POZW 40189 | 54°22’N  22°51’E | NE Poland, Suwałki Lake District, Lake Wiżajny | − | − | − | − | KY706027 | − |  | | |
| H 3 | POZW 42898 | 53°17’N  16°44’E | Central Poland, Wielkopolska, valley of Rurzyca river | KY705623 | KY705817 | KY705893 | KY705715 | KY706028 | KY705505 |  | | |
| H 4 | POZW 42755 | 50°14’N  19°00’E | S Poland, Śląsk, near Katowice | KY705624 | KY705818 | KY705894 | KY705716 | KY706029 | KY705506 |  | | |
| I 1 | POZW 42239 | 54°03’N  18°00’E | NW Poland, Western Pomerania, Lake Małe Oczko | KY705625 | − | − | KY705717 | KY706030 | KY705507 |  | | |
| I 2 | POZW 42768 | 54°03’N  18°00’E | NW Poland, Western Pomerania, Lake Małe Oczko | KY705626 | KY705819 | KY705895 | KY705718 | KY706031 | KY705508 |  | | |
| I 3 | POZW 42760 | 54°03’N  18°00’E | NW Poland, Western Pomerania, Lake Małe Oczko | KY705627 | KY705820 | KY705896 | KY705719 | KY706032 | KY705509 |  | | |
| I 4 | POZW 42899 | 54°16’N  17°29’E | NW Poland, Western Pomerania, Lake Duże Witno | KY705628 | KY705821 | KY705897 | KY705720 | KY706033 | KY705510 |  | | |
| I 5 | POZW 42913 | 54°16’N  17°29’E | NW Poland, Western Pomerania, Lake Duże Witno | KY705629 | KY705822 | − | KY705721 | KY706034 | KY705511 |  | | |
| J 1 | POZW 41053 | 37.86°N  139.76°E | Japan, Mount Lide, N slope | KY705630 | − | − | KY705722 | KY706035 | KY705512 |  | | |
| J 2 | POZW 40544 | 37.86°N  139.76°E | Japan, Mount Lide, N slope | KY705631 | KY705823 | KY705898 | KY705723 | KY706036 | KY705513 |  | | |
| J 3 | POZW 40511 | 37.86°N  139.76°E | Japan, Mount Lide, N slope | KY705632 | KY705824 | KY705899 | KY705724 | KY706037 | KY705514 |  | | |
| J^a^ |  |  | Japan | − | − | − | − | − | DQ986143 |  | | |
| J^c^ |  |  | Dominica | − | − | − | − | FM210488 | − |  | | |
| J^c^ |  |  | Ecuador | − | − | − | − | FM210490 | − |  | | |
| J^e^ |  |  | Japan | − | AB476551 | − | − | − | − |  | | |
| ?^e^ |  |  | Japan | − | AB476550 | − | − | − | − |  | | |
| ?^f^ |  |  | USA | − | AY507391 | − | − | − | − |  | | |
| ***Aneura maxima*** | | | | | | | | | |  | | |
| *A. maxima* 1 | POZW 42612 | no data available | Romania, coll. S. Ştefănut | KY705633 | KY705825 | − | KY705725 | KY706038 | KY705515 |  | | |
| *A. maxima* 2 | POZW 39872 | 52°42’N 23°48’E | E Poland, Białowieża Forest, Żebra Żubra track | KY705634 | KY705826 | − | KY705726 | KY706039 | KY705516 |  | | |
| *A. maxima* 3 | POZW 39882 | 52°41’N 23°53’E | E Poland, Białowieża Forest, Wysokie Bagno Reserve | KY705635 | KY705827 | KY705900 | KY705727 | KY706040 | KY705517 |  | | |
| *A. maxima* 4 | POZW 40324 | 58°48’N 20°24’E | NE Poland, Warmia, Lake Redykajny | KY705636 | KY705828 | KY705901 | KY705728 | KY706041 | KY705518 |  | | |
| *A. maxima* 5 | POZW 41409 | 54°15’N  17°29’E | NW Poland, Western Pomerania, Słupia river | KY705637 | KY705829 | KY705902 | KY705729 | KY706042 | KY705519 |  | | |
| *A. maxima* 6 | POZW 40265 | 53°23’N  16°35’E | Central Poland, Wielkopolska, valley of Rurzyca river | KY705638 | KY705830 | KY705903 | KY705730 | KY706043 | KY705520 |  | | |
| *A. maxima* 7 | POZW 40372 | 53°23’N  16°35’E | Central Poland, Wielkopolska, valley of Rurzyca river | KY705639 | KY705831 | KY705904 | KY705731 | KY706044 | KY705521 |  | | |
| *A. maxima* 8 | POZW 42902 | 49°06’N  22°40’E | SE Poland, Bieszczady Mts, valley of Terebowiec stream | KY705640 | KY705832 | KY705905 | KY705732 | KY706045 | KY705522 |  | | |
| *A. maxima* 9 | POZW 42807 | 49°05’N  22°38’E | SE Poland, Bieszczady Mts, Ustrzyki Górne, small stream | KY705641 | KY705833 | − | − | KY706046 | KY705523 |  | | |
| *A. maxima* 10 | POZW 42820 | 49°26’N  20°27’E | S Poland, Beskidy Mts, valley of Kozłecki stream | KY705642 | KY705834 | KY705906 | KY705733 | KY706047 | KY705524 |  | | |
| *A. maxima* 11 | POZW 40543 | 49°15’N  20°06’E | S Poland, Tatry Mts, Dolina Białki valley | KY705643 | KY705835 | KY705907 | KY705734 | KY706048 | KY705525 |  | | |
| *A. maxima* 12 | POZW 41411 | 49°17’N  20°02’E | S Poland, Las Capowski forest at the foot of Tatry Mts | KY705644 | KY705836 | − | − | KY706049 | KY705526 |  | | |
| *A. maxima* 13 | POZW 42900 | 49°16’N  19°59’E | S Poland, Tatry Mts, Olczyska Valley | KY705645 | KY705837 | − | KY705735 | KY706050 | KY705527 |  | | |
| *A. maxima* 14 | POZW 42438 | 49°34’N  19°33’E | S Poland, Gorce Mts, Działy Orawskie | KY705646 | KY705838 | KY705908 | KY705736 | KY706051 | KY705528 |  | | |
| *A. maxima*^a^ |  |  | USA | − | − | − | − | − | DQ986140 |  | | |
| *A. maxima*^a^ |  |  | Belgium | − | − | − | − | − | DQ986146 |  | | |
| *A. maxima*^b^ |  |  | USA | − | EF547819 | − | − | − | − |  | | |
| *A. maxima*^c^ |  |  | USA | − | − | − | − | FM210483 | − |  | | |
| *A. maxima*^c^ |  |  | Canada | − | − | − | − | FM210484 | − |  | | |
| *A. maxima*^f^ |  |  | USA | − | − | − | − | AY507520 | − |  | | |
| ***A. mirabilis*** | | | | | | | | | |  | | |
| *A. mirabilis*^a^ |  |  | United Kingdom | − | − | − | − | − | DQ986137 |  | | |
| *A. mirabilis*^a^ |  |  | Portugal | − | − | − | − | − | DQ986136 |  | | |
| *A. mirabilis*^b^ |  |  | United Kingdom | − | EF547810 | − | − | − | − |  | | |
| *A. mirabilis*^c^ |  |  | France | − | − | − | − | FM210479 | − |  | | |
| *A. mirabilis*^d^ |  |  | Belgium | − | DQ439683 | − | − | − | − |  | | |

References for sequences taken from GenBank:

^a^ Wickett NJ, Goffinet B (2008) Origin and relationships of the myco-heterotrophic liverwort *Cryptothallus mirabilis* Malmb.(Metzgeriales, Marchantiophyta). *Botanical Journal of the Linnean Society*, **156**, 1–12.

^b^ Wickett NJ, Fan Y, Lewis PO, Goffinet B (2008a) Distribution and evolution of pseudogenes, gene losses, and a gene rearrangement in the plastid genome of the nonphotosynthetic liverwort, *Aneura mirabilis* (Metzgeriales, Jungermanniopsida). *Journal of* Molecular Evolution, **67**,111–122.

^c^ Preussing M, Olsson S, Schäfer-Verwimp A *et al*. (2010) New insights in the evolution of the liverwort family Aneuraceae (Metzgeriales, Marchantiophyta), with on the genus Lobatiriccardia. *Taxon*, **59**, 1424-1440.

^d^ Forrest LL, Davis EC, Long DG, Crandall-Stotler BJ, Clark A, Hollingsworth ML (2006) Unraveling the evolutionary history of the liverworts (Marchantiophyta): multiple taxa, genomes and analyses. *Bryologist* **109**, 303-334.

^e^ Masuzaki H, Shimamura M, Furuki T, Tsubota H, Yamaguchi T, Mohamed MH, Deguchi H (2010) Systematic position of the enigmatic liverwort Mizutania Mizutaniaceae, Marchantiophyta) inferred from molecular phylogenetic analyses. *Taxon*, **59**, 448-458.

^f^ Forrest LL, Crandall-Stotler BJ (2004) A phylogeny of the simple thalloid liverworts (Jungermanniopsida, subclass Metzgeriidae) as inferred from five chloroplast genes. In: *Molecular Systematics of Bryophytes. Monographs in Systematic Botany* (eds. Goffinet B, Hollowell V, Magill R), Missouri Botanical Garden, **98**, 119–140.
