# Supplementary material for: DNA barcoding, ecology and geography of the cryptic species of Aneura pinguis and their relationships with Aneura maxima and Aneura mirabilis (Metzgeriales, Marchantiophyta)
Source: PLoS One. 2017 Dec 5;12(12):e0188837. doi: 10.1371/journal.pone.0188837 (PMC5716573; doi:10.1371/journal.pone.0188837)
Supplement: S2 Table — (DOC) [file pone.0188837.s002.doc]

**S2 Table. Sequences of primers used in the present study.**

| DNA Region | Primer name | Direction | Primer sequence (5’- 3’) | References |
| --- | --- | --- | --- | --- |
| *matK* | RBGE-LIV-F1A | F | TYCATCCWGAAATTTTGATTCG | ^a^ Bell et al. (2012) |
|  | RBGE-LIV-R1A | R | ATAGTACTTTTRTGTTTACATGC |  |
| *rbcL-a* | a_f | F | ATGTCACCACAAACAGAGACTAAAGC | ^b^ Kress & Erickson (2007) |
|  | a_r | R | CTTCTGCTACAAATAAGAATCGATCTC |  |
| *rpoC1* | F | F | TATGAAACCAGAATGGATGG | ^c^ Chase et al. (2007) |
|  | R | R | GAAAACATAAGTARRCGWGC |  |
| *trnH-psbA* | psbA501F | F | TTTCTCAGACGGTATGCC | ^d^ Forrest et al. (2004) |
|  | trnHR | R | GAACGACGGGAATTGAAC |  |
| *trnL-trnF* | A | F | GGGGGTATGGCGAAATTGG | ^e^ Pacak & Szweykowska-Kulińska (2003) |
|  | fR | R | ATTTGAACTGGTGACACGAG | ^f^ Taberlet et al. (1991) |
| ITS1 | F | F | CAAGGTTTCCGTAGGTGAAC | ^g^ Sawicki et al. 2010 |
|  | R | R | CAAGAGCCAAGATATCCG |  |
| ITS2 | F | F | CGGATATCTTGGCTCTTG | ^g^ Sawicki et al. 2010 |
|  | R | R | CCGCTTAGTGATATGCTTA |  |
| PCR cycling conditions: initial denaturation 4 min at 94ºC, followed by 30 cycles 1 min at 94ºC, 30 s at 55 ºC (*matK*) or 62ºC (remaining primers), 1 min at 72ºC, and final extension 7 min at 72ºC. | | | | |

^a^ Bell D, Long DG, Forrest AD *et al.* (2012) DNA barcoding of European *Herbertus* (Marchantiopsida, Herbertaceae) and the discovery and description of a new species. *Molecular Ecology Resources*, **12**, 36–47.

^b^ Kress WJ, Erickson DL (2007) A two-locus global DNA barcode for land plants: the coding rbcL gene complements the non-coding trnH-psbA spacer region. *Public Library of Science, ONE*, **2**, e508.

^c^ Chase MW, Cowan RS, Hollingsworth PM *et al*. (2007) A proposal for a standardised protocol to barcode all land plants. *Taxon*, **56**, 295–299.

^d^ Forrest LL, Crandall-Stotler BJ (2004) A phylogeny of the simple thalloid liverworts (Jungermanniopsida, subclass Metzgeriidae) as inferred from five chloroplast genes. In: *Molecular Systematics of Bryophytes. Monographs in Systematic Botany* (eds Goffinet B, Hollowell V, Magill R), Missouri Botanical Garden, **98**, 119–140.

^e^ Pacak A, Szweykowska-Kulińska Z (2003) Organellar inheritance in liverworts: an example of *Pellia borealis*. *Journal of Molecular Evolution*, **56**, 11-17.

^f^ Taberlet P, Gielly L, Pautou G, Bouvet J (1991) Universal primers for amplification of three non-coding regions of chloroplast DNA. *Plant Molecular Biology*, **17**, 1105–1109.

^g^ Sawicki J, Plášek V, Szczecińska M (2010) Molecular studies resolve *Nyholmiella* (Orthotrichaceae) as a separate genus. *Journal of Systematics and Evolution*, **48**, 183-194.
