# Supplementary material for: DNA barcoding, ecology and geography of the cryptic species of Aneura pinguis and their relationships with Aneura maxima and Aneura mirabilis (Metzgeriales, Marchantiophyta)
Source: PLoS One. 2017 Dec 5;12(12):e0188837. doi: 10.1371/journal.pone.0188837 (PMC5716573; doi:10.1371/journal.pone.0188837)
Supplement: S3 Table — (DOC) [file pone.0188837.s003.doc]

**S3** **Table. Average genetic divergences (K2P %) for *A. pinguis* cryptic species, *A. maxima* and *A. mirabilis*;** combined plastid sequences (below diagonal) and ITS (above diagonal).

|  | A | B | C | D | E | F | G | H | I | J | *A. maxima* | *A. mirabilis* |
| --- | --- | --- | --- | --- | --- | --- | --- | --- | --- | --- | --- | --- |
| A | *** | 10.48 | 10.13 | 9.06 | 9.18 | 10.47 | 9.36 | 11.38 | 9.46 | 9.25 | 8.86 | 7.99 |
| B | 5.12 | *** | 2.63 | 12.40 | 12.57 | 1.56 | 7.38 | 7.06 | 8.42 | 11.94 | 8.89 | 7.89 |
| C | 4.96 | 1.22 | *** | 11.76 | 11.94 | 2.50 | 6.86 | 7.81 | 7.87 | 11.19 | 7.54 | 7.47 |
| D | 3.86 | 5.72 | 5.72 | *** | 4.84 | 12.79 | 10.01 | 11.46 | 10.01 | 5.42 | 9.03 | 8.46 |
| E | 3.57 | 5.47 | 5.36 | 2.15 | *** | 12.97 | 9.70 | 12.18 | 9.46 | 5.25 | 8.56 | 8.67 |
| F | 5.56 | 1.44 | 1.80 | 6.31 | 6.13 | *** | 7.34 | 7.36 | 8.46 | 12.21 | 9.01 | 8.15 |
| G | 4.46 | 2.85 | 2.89 | 5.35 | 4.95 | 3.40 | *** | 6.12 | 7.83 | 8.88 | 8.37 | 7.54 |
| H | 5.07 | 2.98 | 3.01 | 5.65 | 5.06 | 3.66 | 1.77 | *** | 9.43 | 11.01 | 9.36 | 7.86 |
| I | 4.53 | 3.53 | 3.39 | 5.10 | 4.95 | 4.04 | 3.02 | 3.27 | *** | 10.05 | 7.28 | 4.81 |
| J | 3.63 | 5.95 | 5.89 | 2.74 | 2.03 | 6.38 | 5.39 | 5.72 | 5.20 | *** | 9.62 | 8.50 |
| *A. maxima* | 4.47 | 2.676 | 2.53 | 4.93 | 4.54 | 3.13 | 2.51 | 2.65 | 2.64 | 5.06 | *** | 5.34 |
| *A. mirabilis* | 4.98 | 3.529 | 3.45 | 5.72 | 5.16 | 4.09 | 3.37 | 3.54 | 2.93 | 5.56 | 2.67 | *** |
