# Supplementary material for: DNA barcoding, ecology and geography of the cryptic species of Aneura pinguis and their relationships with Aneura maxima and Aneura mirabilis (Metzgeriales, Marchantiophyta)
Source: PLoS One. 2017 Dec 5;12(12):e0188837. doi: 10.1371/journal.pone.0188837 (PMC5716573; doi:10.1371/journal.pone.0188837)
Supplement: S4 Table — (DOC) [file pone.0188837.s004.doc]

**S4** **Table**. **Average genetic divergences (K2P %) for *A. pinguis* lineages, *A. maxima* and *A. mirabilis***; combined plastid sequences (below diagonal) and ITS (above diagonal).

|  | A1 | A2 | A3 | B1 | B2 | B3 | C1 | C2 | D | E1 | E2 | F | G | H | I | J | *A. maxima* | *A. mirabilis* |
| --- | --- | --- | --- | --- | --- | --- | --- | --- | --- | --- | --- | --- | --- | --- | --- | --- | --- | --- |
| A1 | *** | 0.21 | 2.63 | 10.83 | 10.85 | 11.57 | 10.39 | 10.63 | 9.81 | 10.00 | 10.14 | 10.94 | 9.77 | 11.88 | 10.10 | 10.18 | 9.59 | 8.69 |
| A2 | 0.19 | *** | 2.41 | 10.01 | 10.03 | 9.92 | 9.89 | 10.12 | 8.40 | 8.41 | 8.86 | 10.12 | 8.98 | 10.92 | 8.97 | 8.44 | 8.29 | 7.45 |
| A3 | 0.68 | 0.66 | *** | 9.80 | 9.82 | 10.03 | 9.68 | 9.91 | 8.48 | 8.49 | 8.94 | 9.91 | 9.09 | 11.03 | 8.77 | 8.52 | 8.12 | 7.25 |
| B1 | 5.12 | 5.05 | 5.05 | *** | 0.95 | 1.57 | 2.64 | 2.85 | 12.41 | 12.56 | 13.08 | 1.85 | 7.53 | 6.97 | 8.13 | 12.12 | 8.90 | 7.76 |
| B2 | 5.13 | 5.06 | 5.06 | 0.26 | *** | 1.03 | 2.37 | 2.58 | 12.37 | 12.52 | 13.03 | 1.06 | 7.26 | 6.98 | 8.39 | 11.80 | 8.61 | 7.78 |
| B3 | 5.27 | 5.20 | 5.19 | 0.61 | 0.47 | *** | 2.72 | 2.93 | 12.42 | 12.54 | 13.05 | 1.67 | 7.32 | 7.20 | 8.73 | 11.86 | 9.11 | 8.12 |
| C1 | 4.94 | 5.00 | 4.99 | 1.18 | 1.15 | 1.51 | *** | 0.62 | 11.72 | 11.87 | 12.38 | 2.44 | 6.79 | 7.77 | 7.78 | 11.15 | 7.51 | 7.48 |
| C2 | 4.93 | 4.91 | 4.90 | 1.06 | 1.03 | 1.39 | 0.23 | *** | 11.96 | 12.11 | 12.62 | 2.81 | 7.19 | 7.99 | 8.32 | 11.40 | 7.72 | 7.39 |
| D | 3.97 | 3.77 | 3.78 | 5.65 | 5.68 | 5.86 | 5.74 | 5.58 | *** | 4.83 | 4.95 | 12.79 | 10.01 | 11.46 | 10.01 | 5.42 | 9.03 | 8.46 |
| E1 | 3.59 | 3.54 | 3.57 | 5.42 | 5.42 | 5.68 | 5.39 | 5.32 | 2.14 | *** | 0.64 | 12.94 | 9.68 | 12.15 | 9.44 | 5.24 | 8.53 | 8.64 |
| E2 | 3.53 | 3.54 | 3.57 | 5.42 | 5.42 | 5.62 | 5.29 | 5.20 | 2.17 | 0.33 | *** | 13.46 | 10.02 | 12.62 | 9.85 | 5.34 | 9.02 | 9.09 |
| F | 5.58 | 5.57 | 5.53 | 1.39 | 1.39 | 1.63 | 1.82 | 1.70 | 6.31 | 6.14 | 6.09 | *** | 7.34 | 7.36 | 8.46 | 12.21 | 9.01 | 8.15 |
| G | 4.47 | 4.47 | 4.43 | 2.80 | 2.80 | 3.05 | 2.92 | 2.74 | 5.35 | 4.94 | 5.00 | 3.40 | *** | 6.12 | 7.83 | 8.88 | 8.37 | 7.54 |
| H | 5.05 | 5.10 | 5.09 | 2.92 | 2.92 | 3.17 | 3.02 | 2.90 | 5.65 | 5.04 | 5.16 | 3.66 | 1.77 | *** | 9.43 | 11.01 | 9.36 | 7.86 |
| I | 4.47 | 4.59 | 4.55 | 3.48 | 3.48 | 3.72 | 3.40 | 3.31 | 5.10 | 4.94 | 4.98 | 4.04 | 3.02 | 3.27 | *** | 10.05 | 7.28 | 4.81 |
| J | 3.64 | 3.62 | 3.61 | 5.93 | 5.87 | 6.13 | 5.90 | 5.81 | 2.74 | 2.024 | 2.05 | 6.38 | 5.39 | 5.72 | 5.20 | *** | 9.62 | 8.50 |
| *A. maxima* | 4.48 | 4.47 | 4.46 | 2.65 | 2.59 | 2.90 | 2.55 | 2.46 | 4.93 | 4.55 | 4.51 | 3.13 | 2.51 | 2.65 | 2.64 | 5.06 | *** | 5.34 |
| *A. mirabilis* | 5.01 | 5.02 | 4.88 | 3.47 | 3.47 | 3.73 | 3.47 | 3.35 | 5.72 | 5.18 | 5.09 | 4.09 | 3.37 | 3.54 | 2.93 | 5.56 | 2.67 | *** |
