# Supplementary figures and images for: DNA barcoding, ecology and geography of the cryptic species of Aneura pinguis and their relationships with Aneura maxima and Aneura mirabilis (Metzgeriales, Marchantiophyta)
Source: PLoS One. 2017 Dec 5;12(12):e0188837. doi: 10.1371/journal.pone.0188837 (PMC5716573; doi:10.1371/journal.pone.0188837)

A

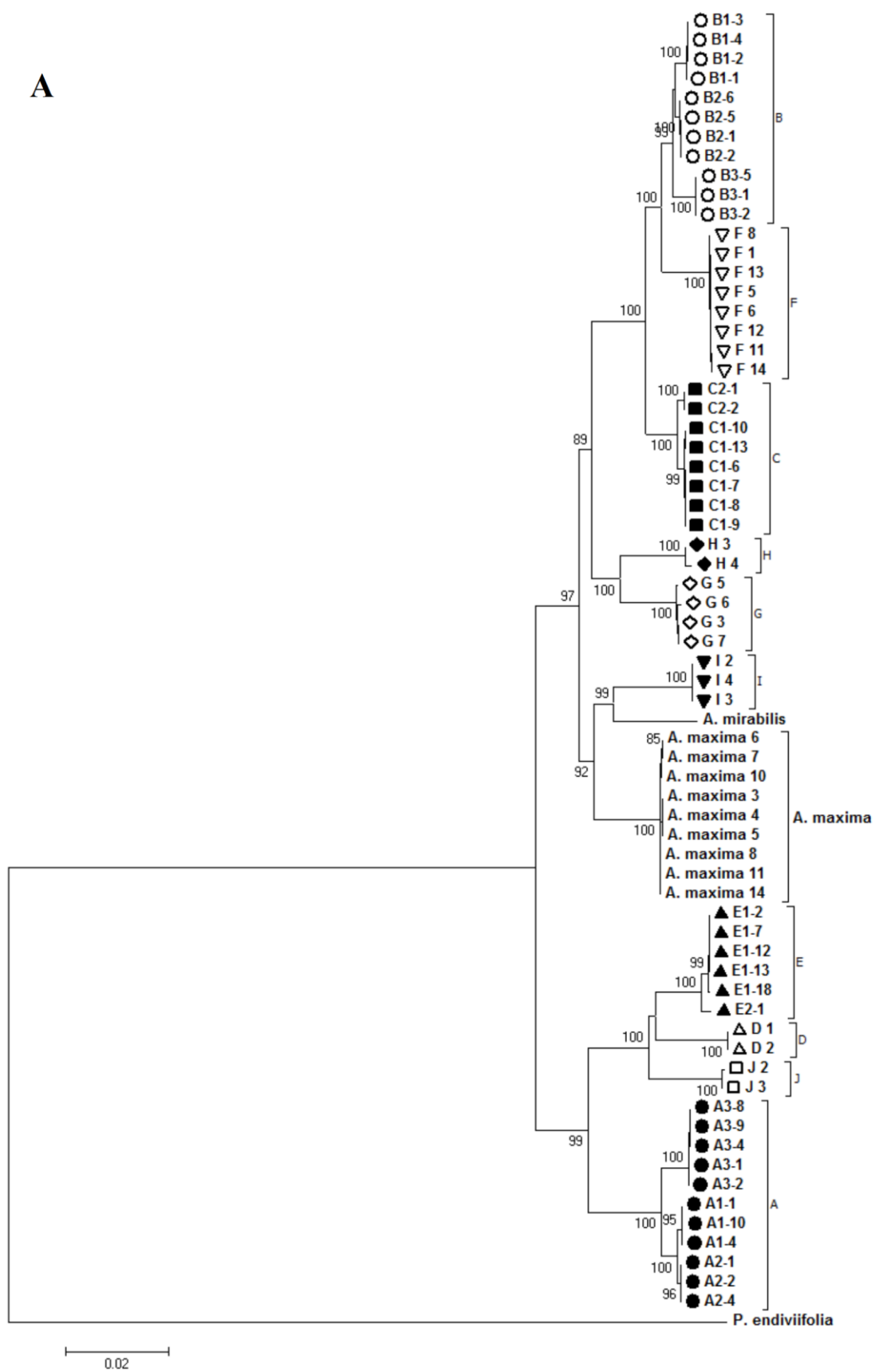

**B**

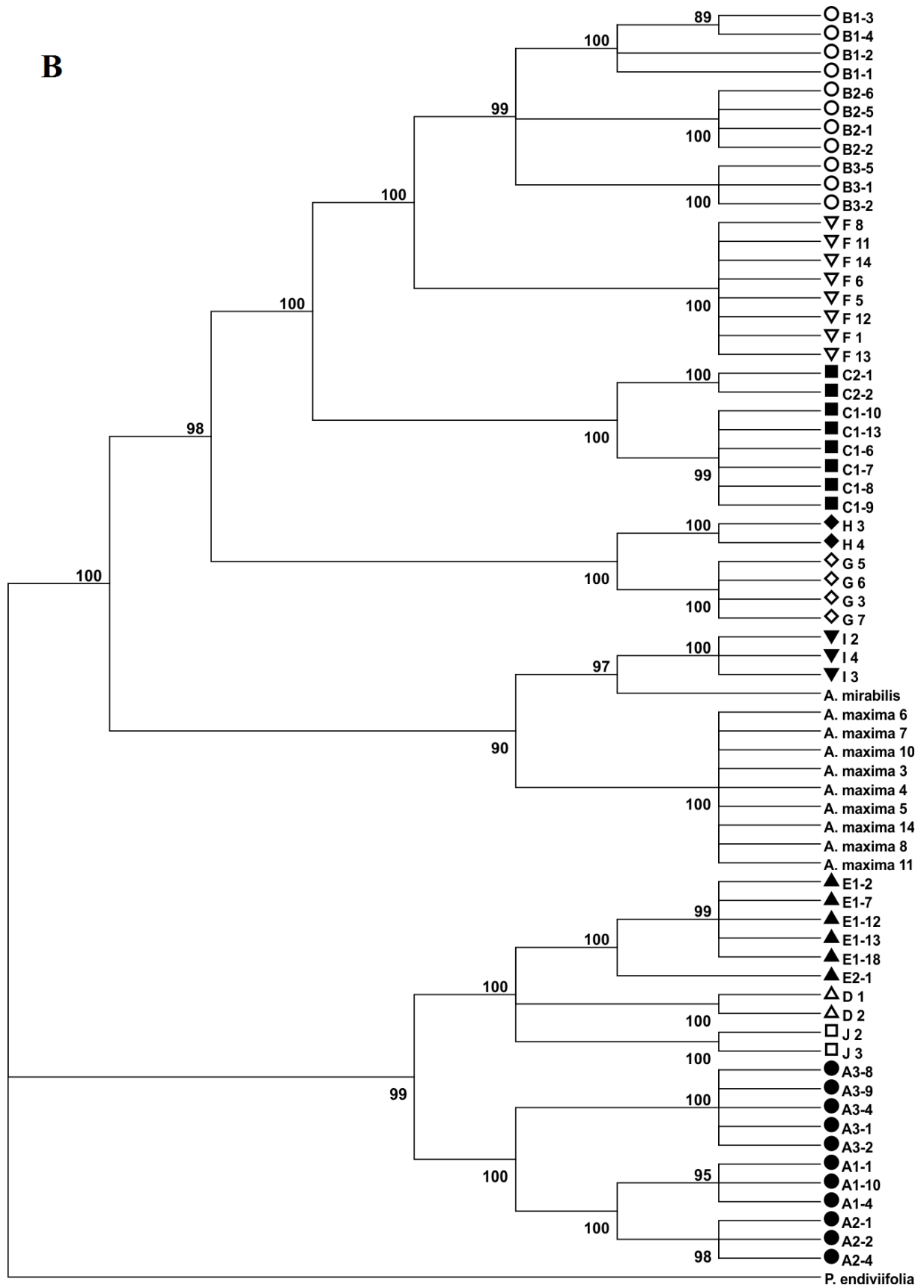

Supplement: S1 Fig — (PDF) [file pone.0188837.s005.pdf]

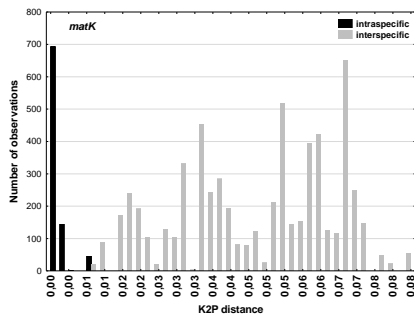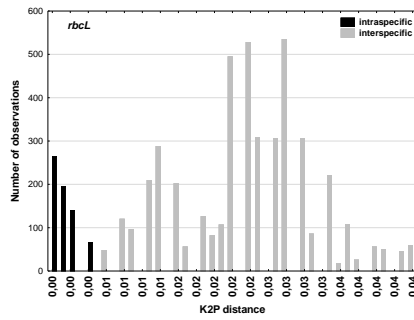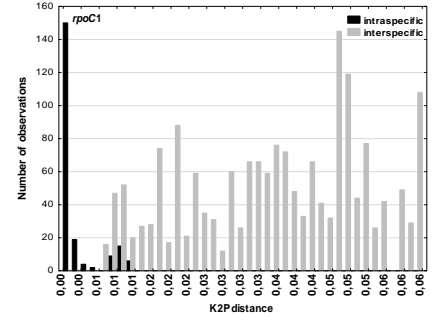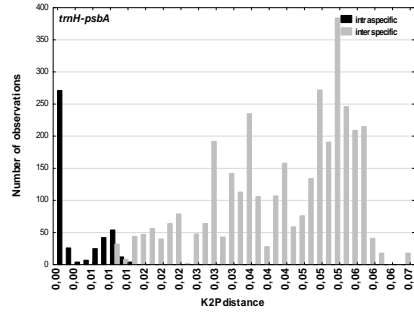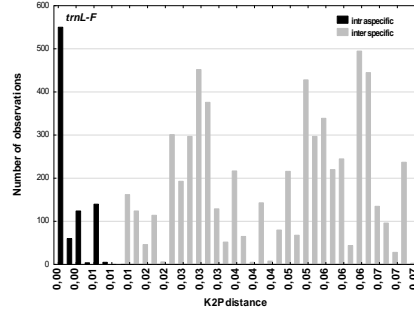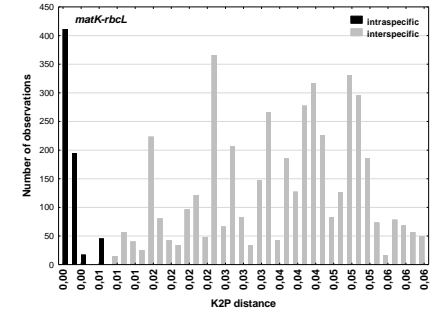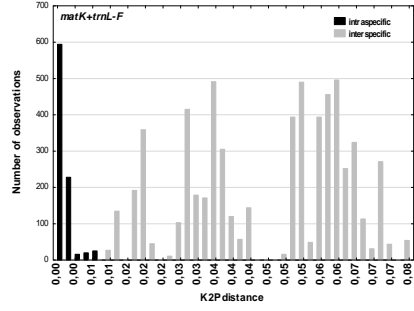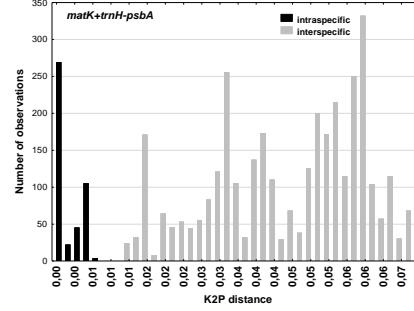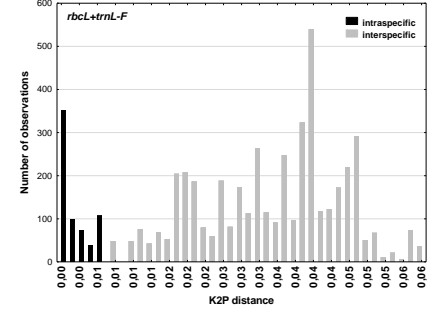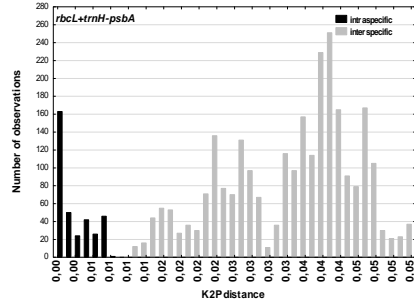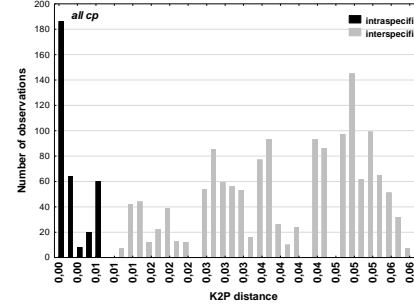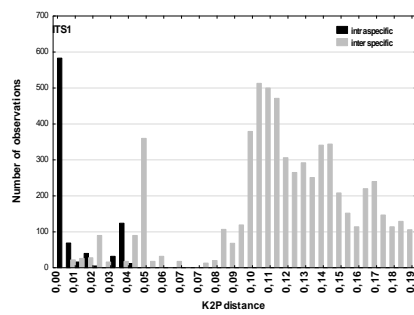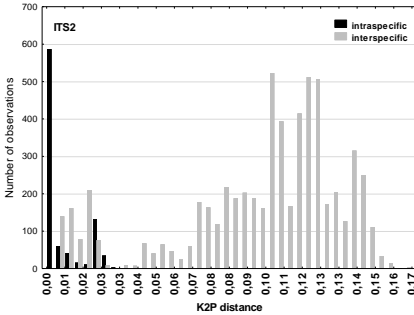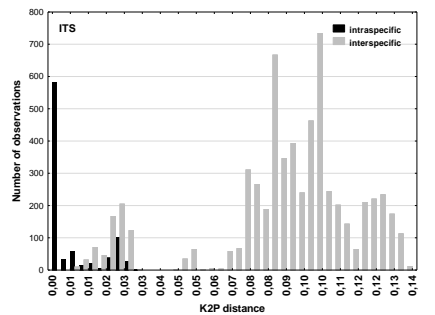

Supplement: S3 Fig — (PDF) [file pone.0188837.s007.pdf]
