# Supplementary material for: DNA barcoding, ecology and geography of the cryptic species of Aneura pinguis and their relationships with Aneura maxima and Aneura mirabilis (Metzgeriales, Marchantiophyta)
Source: PLoS One. 2017 Dec 5;12(12):e0188837. doi: 10.1371/journal.pone.0188837 (PMC5716573; doi:10.1371/journal.pone.0188837)
Supplement: S2 Fig — Neighbor joining (A) and maximum parsimony (B) consensus trees of Aneura. pinguis cryptic species based on a combined dataset. Aneura maxima and A. mirabilis were used for comparison. Pellia endiviifolia was used as an outgroup. Only the accessions with the sequences obtained for all loci were included in the analysis. Bootstrap values above 85% are indicated above branches. (PDF) [file pone.0188837.s006.pdf]

S2 Fig. **Neighbor-joining 75% majority-rule bootstrap consensus trees for the studied species of *Aneura* genus.**

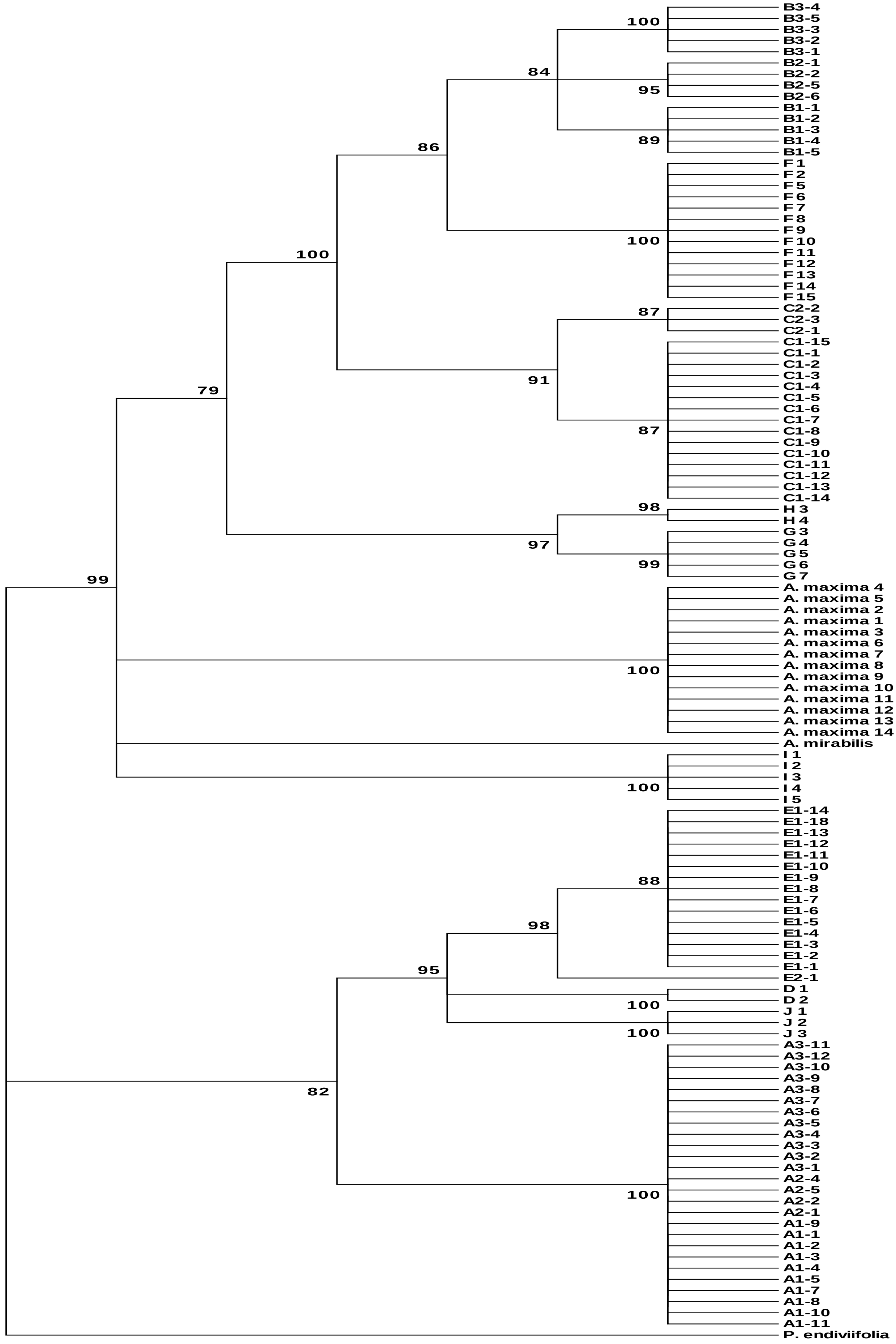

matK

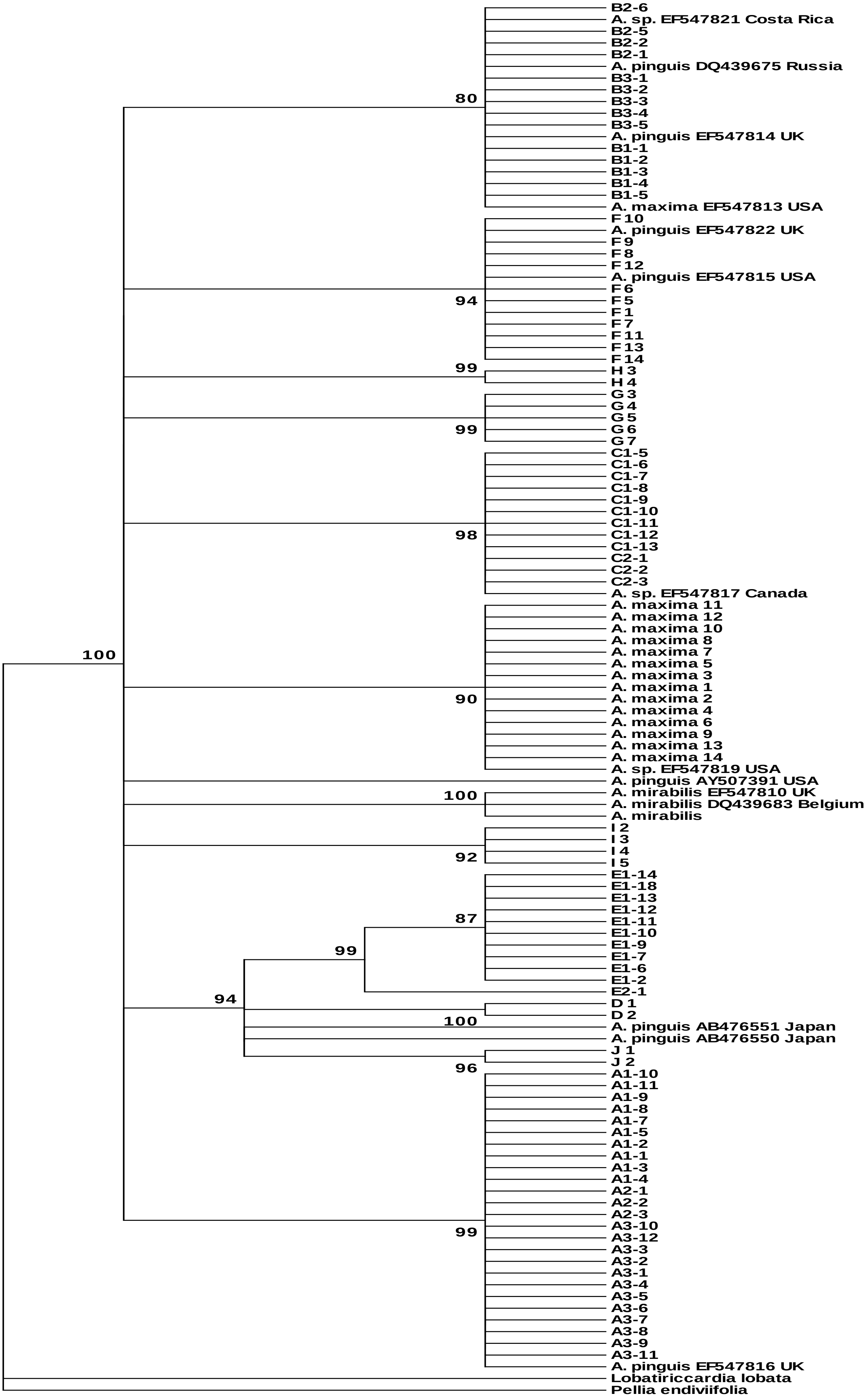
B2-6
A. sp. EF547821 Costa Rica
B2-5
B2-2
B2-1
A. pinguis DQ439675 Russia
B3-1
B3-2
B3-3
B3-4
B3-5
A. pinguis EF547814 UK
B1-1
B1-2
B1-3
B1-4
B1-5
A. maxima EF547813 USA
F 10
A. pinguis EF547822 UK
F 9
F 8
F 12
A. pinguis EF547815 USA
F 6
F 5
F 1
F 7
F 11
F 13
F 14
H 3
H 4
G 3
G 4
G 5
G 6
G 7
C1-5
C1-6
C1-7
C1-8
C1-9
C1-10
C1-11
C1-12
C1-13
C2-1
C2-2
C2-3
A. sp. EF547817 Canada
A. maxima 11
A. maxima 12
A. maxima 10
A. maxima 8
A. maxima 7
A. maxima 5
A. maxima 3
A. maxima 1
A. maxima 2
A. maxima 4
A. maxima 6
A. maxima 9
A. maxima 13
A. maxima 14
A. sp. EF547819 USA
A. pinguis AY507391 USA
A. mirabilis EF547810 UK
A. mirabilis DQ439683 Belgium
A. mirabilis
I 2
I 3
I 4
I 5
E1-14
E1-18
E1-13
E1-12
E1-11
E1-10
E1-9
E1-7
E1-6
E1-2
E2-1
D 1
D 2
A. pinguis AB476551 Japan
A. pinguis AB476550 Japan
J 1
J 2
A1-10
A1-11
A1-9
A1-8
A1-7
A1-5
A1-2
A1-1
A1-3
A1-4
A2-1
A2-2
A2-3
A3-10
A3-12
A3-3
A3-2
A3-1
A3-4
A3-5
A3-6
A3-7
A3-8
A3-9
A3-11
A. pinguis EF547816 UK
Lobatiriccardia lobata
Pellia endiviifolia
80
94
99
99
98
100
90
100
92
87
99
94
100
96
99

rbcL

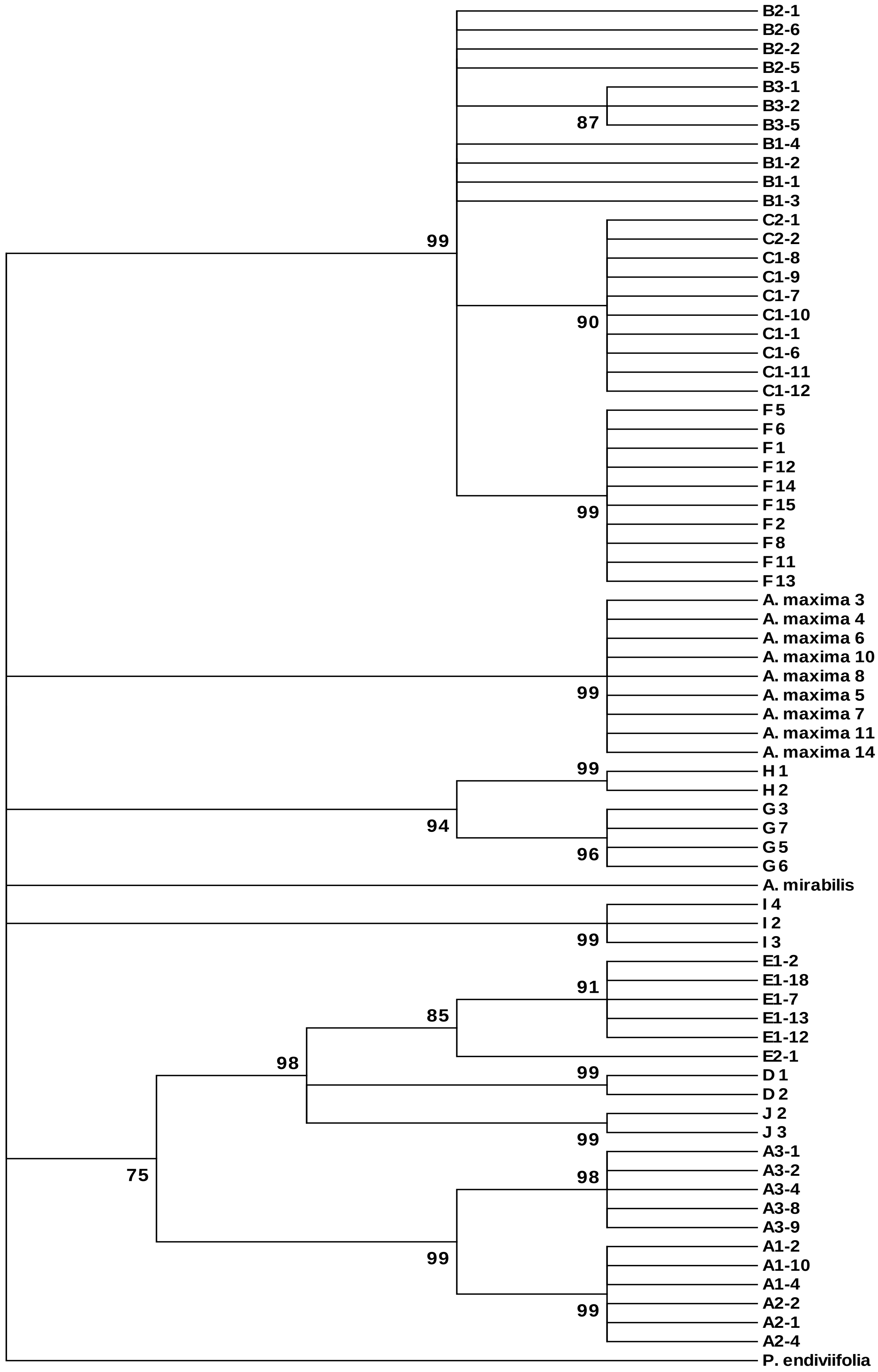
B2-1
B2-6
B2-2
B2-5
B3-1
B3-2
B3-5
87
B1-4
B1-2
B1-1
B1-3
99
C2-1
C2-2
C1-8
C1-9
C1-7
C1-10
90
C1-1
C1-6
C1-11
C1-12
F 5
F 6
F 1
F 12
F 14
F 15
99
F 2
F 8
F 11
F 13
A. maxima 3
A. maxima 4
A. maxima 6
A. maxima 10
A. maxima 8
99
A. maxima 5
A. maxima 7
A. maxima 11
A. maxima 14
99
H 1
H 2
94
G 3
G 7
96
G 5
G 6
A. mirabilis
I 4
I 2
99
I 3
E1-2
E1-18
91
E1-7
85
E1-13
E1-12
E2-1
98
99
D 1
D 2
J 2
J 3
99
A3-1
A3-2
98
A3-4
75
A3-8
A3-9
A1-2
99
A1-10
A1-4
A2-2
99
A2-1
A2-4
P. endiviifolia

rpoC1

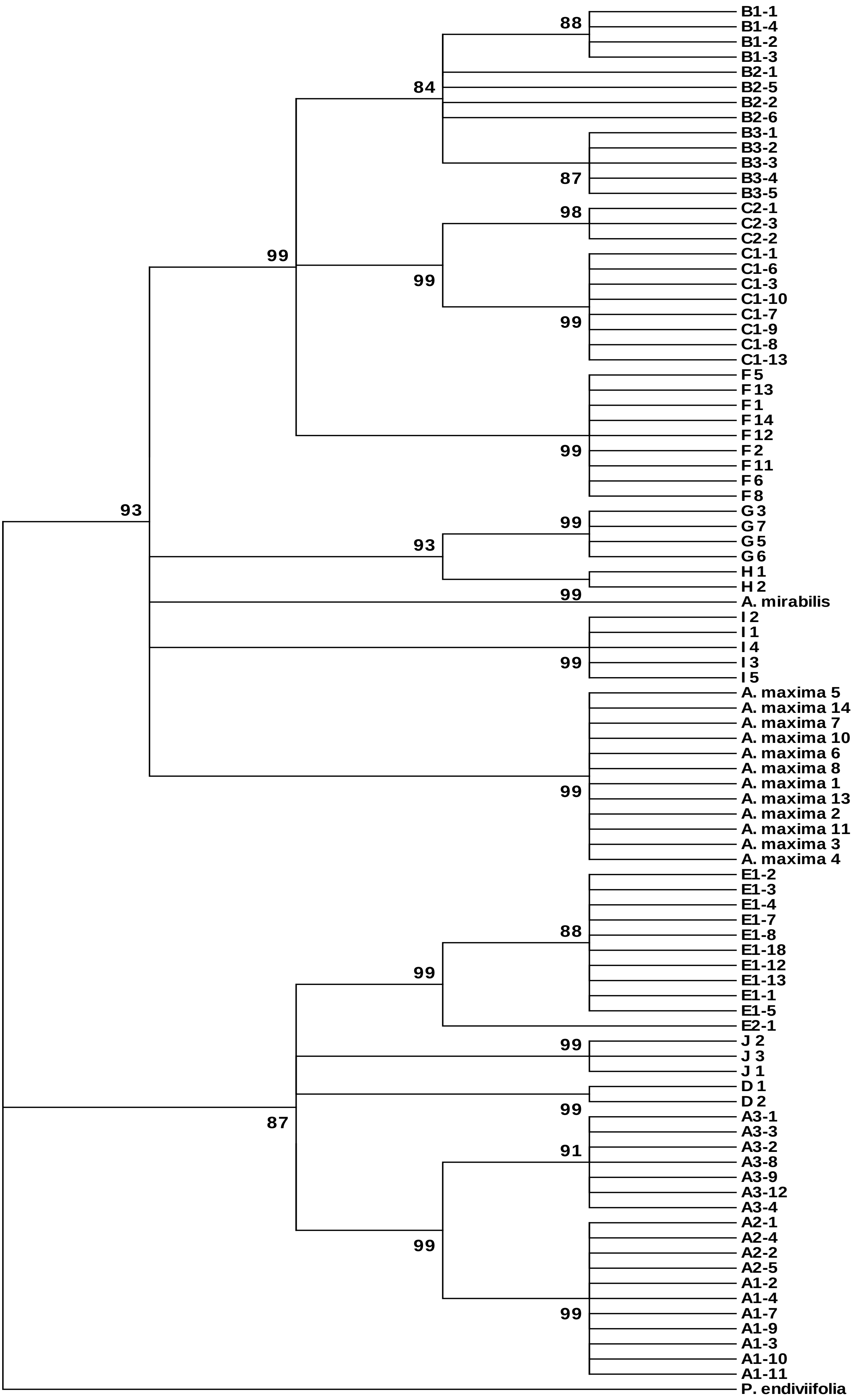
B1-1
B1-4
B1-2
B1-3
B2-1
B2-5
B2-2
B2-6
B3-1
B3-2
B3-3
B3-4
B3-5
C2-1
C2-3
C2-2
C1-1
C1-6
C1-3
C1-10
C1-7
C1-9
C1-8
C1-13
F 5
F 13
F 1
F 14
F 12
F 2
F 11
F 6
F 8
G 3
G 7
G 5
G 6
H 1
H 2
A. mirabilis
I 2
I 1
I 4
I 3
I 5
A. maxima 5
A. maxima 14
A. maxima 7
A. maxima 10
A. maxima 6
A. maxima 8
A. maxima 1
A. maxima 13
A. maxima 2
A. maxima 11
A. maxima 3
A. maxima 4
E1-2
E1-3
E1-4
E1-7
E1-8
E1-18
E1-12
E1-13
E1-1
E1-5
E2-1
J 2
J 3
J 1
D 1
D 2
A3-1
A3-3
A3-2
A3-8
A3-9
A3-12
A3-4
A2-1
A2-4
A2-2
A2-5
A1-2
A1-4
A1-7
A1-9
A1-3
A1-10
A1-11
P. endiviifolia
88
84
87
98
99
99
99
99
93
99
93
99
99
99
88
99
99
87
99
91
99
99

trnH-psbA

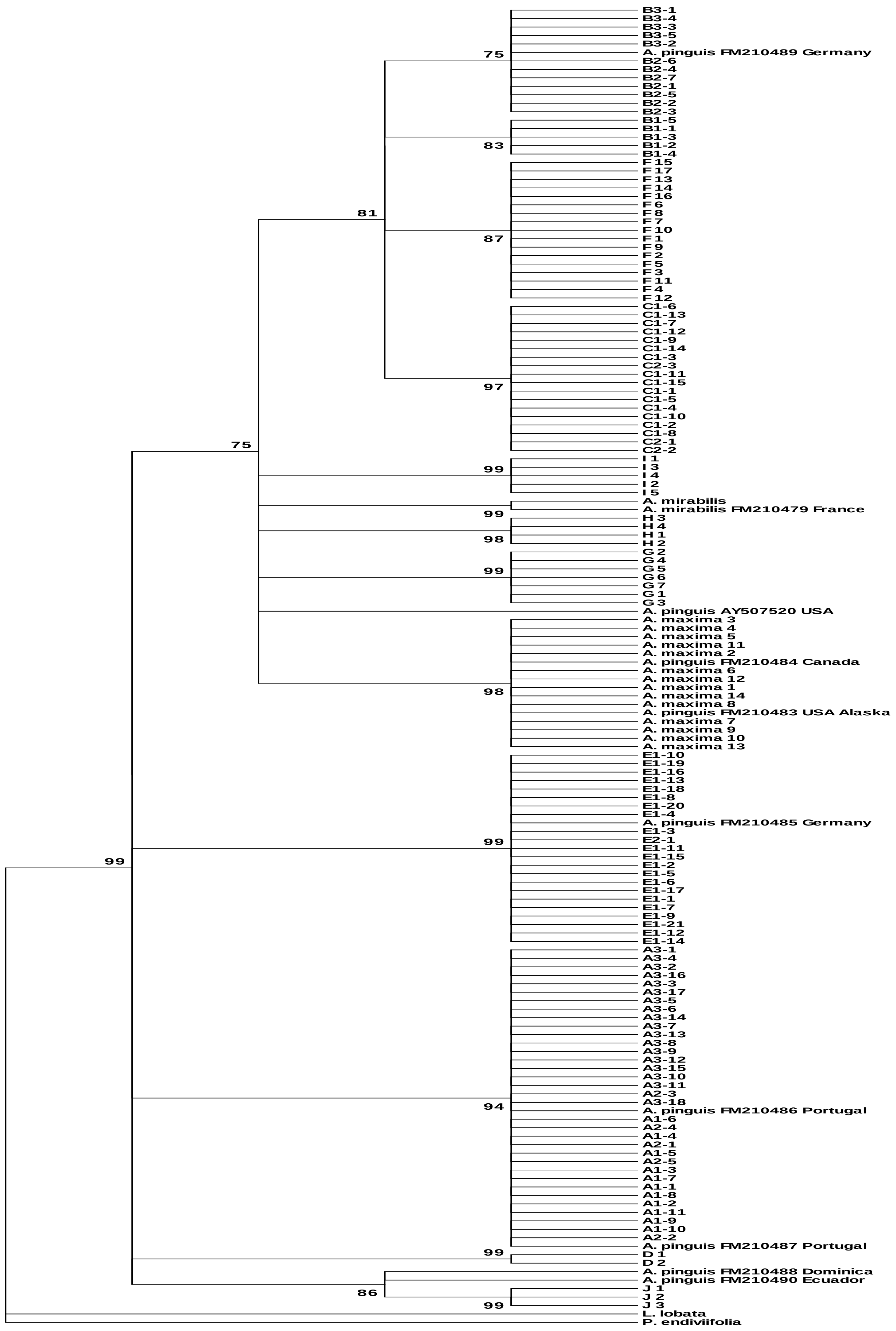
B3-1
B3-4
B3-3
B3-5
B3-2
A. pinguis FM210489 Germany
B2-6
B2-4
B2-7
B2-1
B2-5
B2-2
B2-3
B1-5
B1-1
B1-3
B1-2
B1-4
F15
F17
F13
F14
F16
F6
F8
F7
F10
F1
F9
F2
F5
F3
F11
F4
F12
C1-6
C1-13
C1-7
C1-12
C1-9
C1-14
C1-3
C2-3
C1-11
C1-15
C1-1
C1-5
C1-4
C1-10
C1-2
C1-8
C2-1
C2-2
I1
I3
I4
I2
I5
A. mirabilis
A. mirabilis FM210479 France
H3
H4
H1
H2
G2
G4
G5
G6
G7
G1
G3
A. pinguis AY507520 USA
A. maxima 3
A. maxima 4
A. maxima 5
A. maxima 11
A. maxima 2
A. pinguis FM210484 Canada
A. maxima 6
A. maxima 12
A. maxima 1
A. maxima 14
A. maxima 8
A. pinguis FM210483 USA Alaska
A. maxima 7
A. maxima 9
A. maxima 10
A. maxima 13
E1-10
E1-19
E1-16
E1-13
E1-18
E1-8
E1-20
E1-4
A. pinguis FM210485 Germany
E1-3
E2-1
E1-11
E1-15
E1-2
E1-5
E1-6
E1-17
E1-1
E1-7
E1-9
E1-21
E1-12
E1-14
A3-1
A3-4
A3-2
A3-16
A3-3
A3-17
A3-5
A3-6
A3-14
A3-7
A3-13
A3-8
A3-9
A3-12
A3-15
A3-10
A3-11
A2-3
A3-18
A. pinguis FM210486 Portugal
A1-6
A2-4
A1-4
A2-1
A1-5
A2-5
A1-3
A1-7
A1-1
A1-8
A1-2
A1-11
A1-9
A1-10
A2-2
A. pinguis FM210487 Portugal
D1
D2
A. pinguis FM210488 Dominica
A. pinguis FM210490 Ecuador
J1
J2
J3
L. lobata
P. endiviifolia
75
83
81
87
97
75
99
99
98
99
98
99
99
94
99
86
99

trnL-trnF

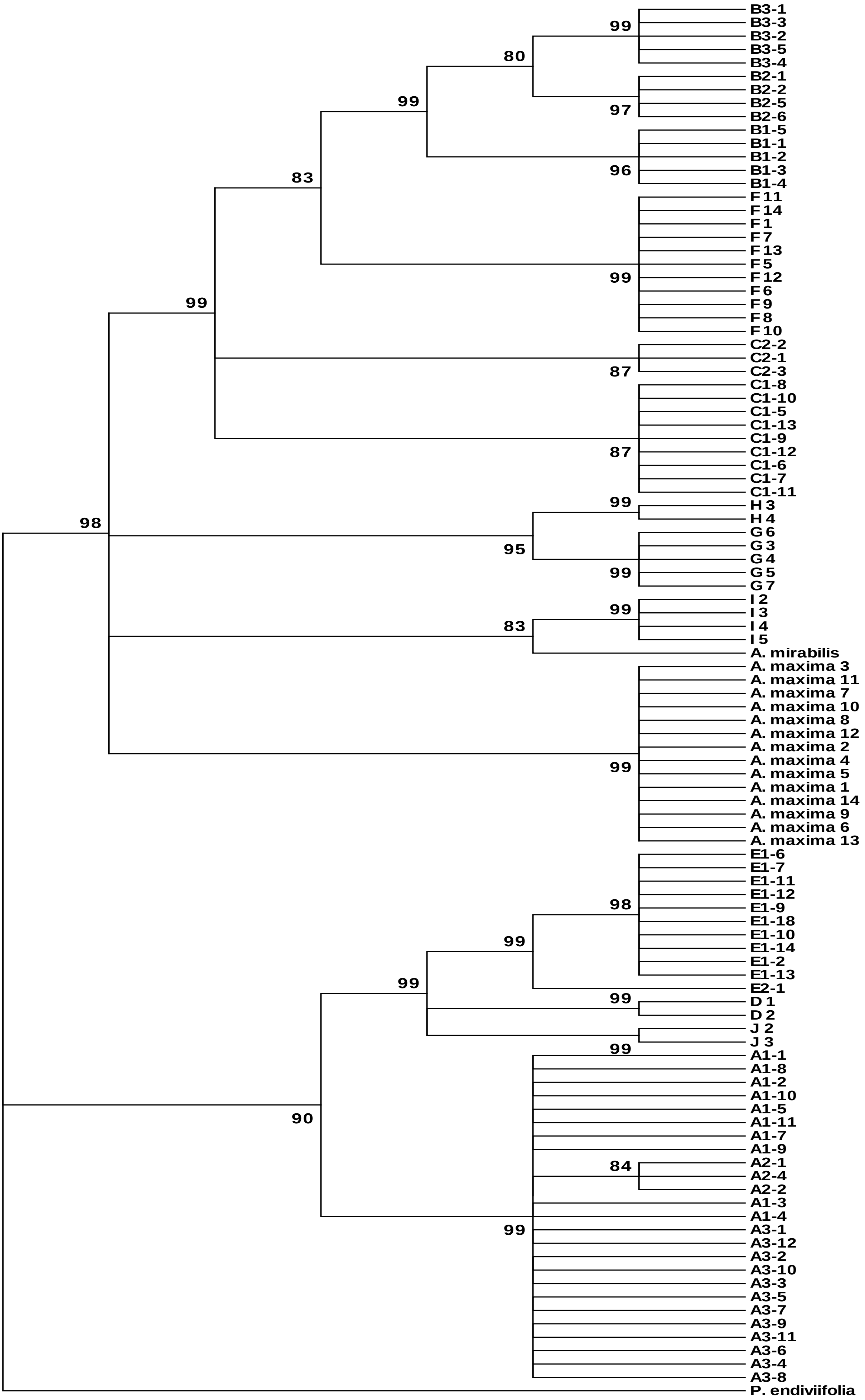
B3-1
B3-3
B3-2
B3-5
B3-4
B2-1
B2-2
B2-5
B2-6
B1-5
B1-1
B1-2
B1-3
B1-4
F 11
F 14
F 1
F 7
F 13
F 5
F 12
F 6
F 9
F 8
F 10
C2-2
C2-1
C2-3
C1-8
C1-10
C1-5
C1-13
C1-9
C1-12
C1-6
C1-7
C1-11
H 3
H 4
G 6
G 3
G 4
G 5
G 7
I 2
I 3
I 4
I 5
A. mirabilis
A. maxima 3
A. maxima 11
A. maxima 7
A. maxima 10
A. maxima 8
A. maxima 12
A. maxima 2
A. maxima 4
A. maxima 5
A. maxima 1
A. maxima 14
A. maxima 9
A. maxima 6
A. maxima 13
E1-6
E1-7
E1-11
E1-12
E1-9
E1-18
E1-10
E1-14
E1-2
E1-13
E2-1
D 1
D 2
J 2
J 3
A1-1
A1-8
A1-2
A1-10
A1-5
A1-11
A1-7
A1-9
A2-1
A2-4
A2-2
A1-3
A1-4
A3-1
A3-12
A3-2
A3-10
A3-3
A3-5
A3-7
A3-9
A3-11
A3-6
A3-4
A3-8
P. endiviifolia
99
80
97
99
83
96
99
99
87
87
98
99
95
99
99
83
99
98
99
99
99
99
90
84
99

matK+rbcL

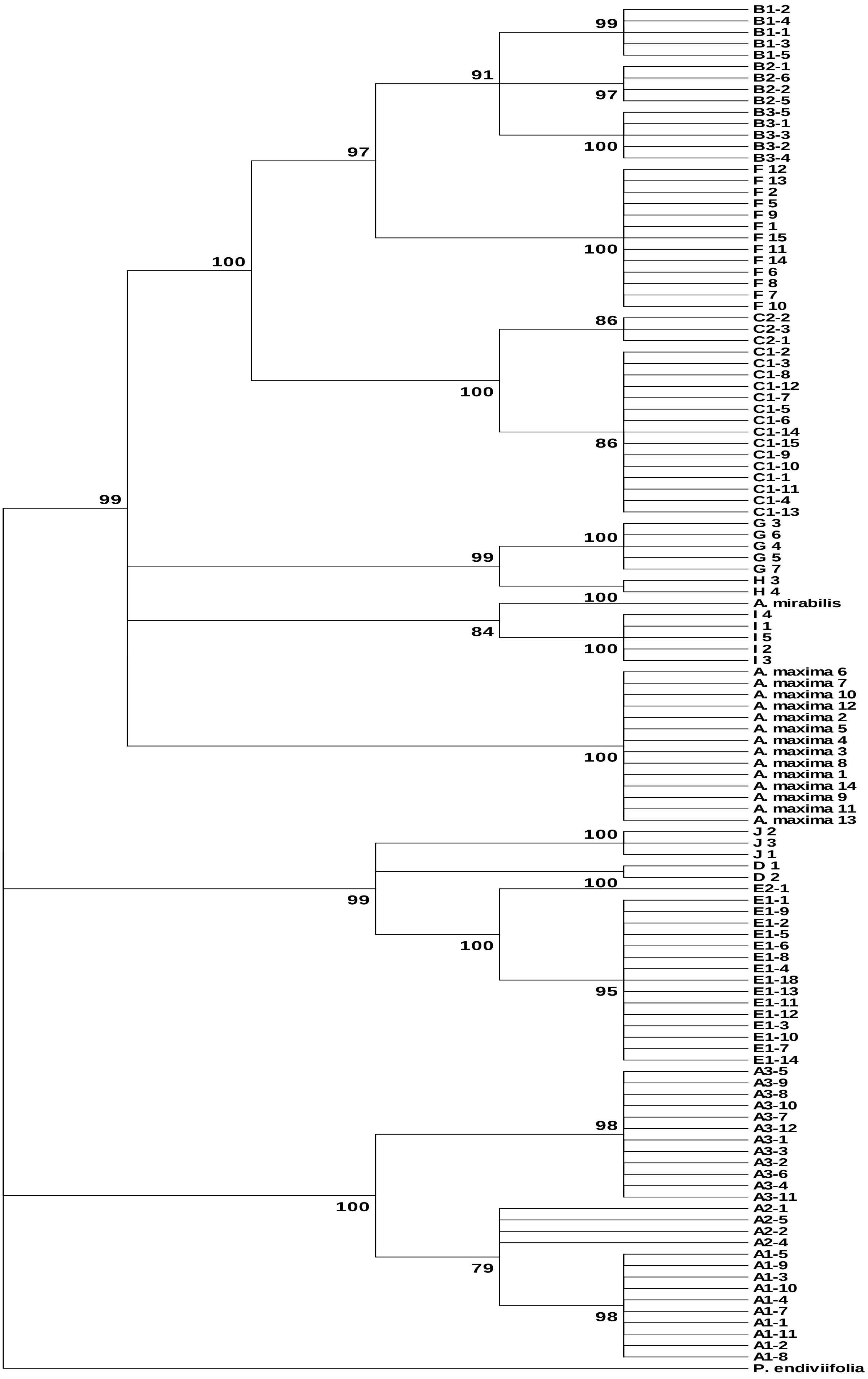
B1-2
B1-4
B1-1
B1-3
B1-5
B2-1
B2-6
B2-2
B2-5
B3-5
B3-1
B3-3
B3-2
B3-4
F 12
F 13
F 2
F 5
F 9
F 1
F 15
F 11
F 14
F 6
F 8
F 7
F 10
C2-2
C2-3
C2-1
C1-2
C1-3
C1-8
C1-12
C1-7
C1-5
C1-6
C1-14
C1-15
C1-9
C1-10
C1-1
C1-11
C1-4
C1-13
G 3
G 6
G 4
G 5
G 7
H 3
H 4
A. mirabilis
I 4
I 1
I 5
I 2
I 3
A. maxima 6
A. maxima 7
A. maxima 10
A. maxima 12
A. maxima 2
A. maxima 5
A. maxima 4
A. maxima 3
A. maxima 8
A. maxima 1
A. maxima 14
A. maxima 9
A. maxima 11
A. maxima 13
J 2
J 3
J 1
D 1
D 2
E2-1
E1-1
E1-9
E1-2
E1-5
E1-6
E1-8
E1-4
E1-18
E1-13
E1-11
E1-12
E1-3
E1-10
E1-7
E1-14
A3-5
A3-9
A3-8
A3-10
A3-7
A3-12
A3-1
A3-3
A3-2
A3-6
A3-4
A3-11
A2-1
A2-5
A2-2
A2-4
A1-5
A1-9
A1-3
A1-10
A1-4
A1-7
A1-1
A1-11
A1-2
A1-8
P. endiviifolia
99
97
91
100
97
100
100
86
100
86
99
100
99
100
84
100
100
100
100
99
100
100
95
98
100
79
98

matK+trnL-F

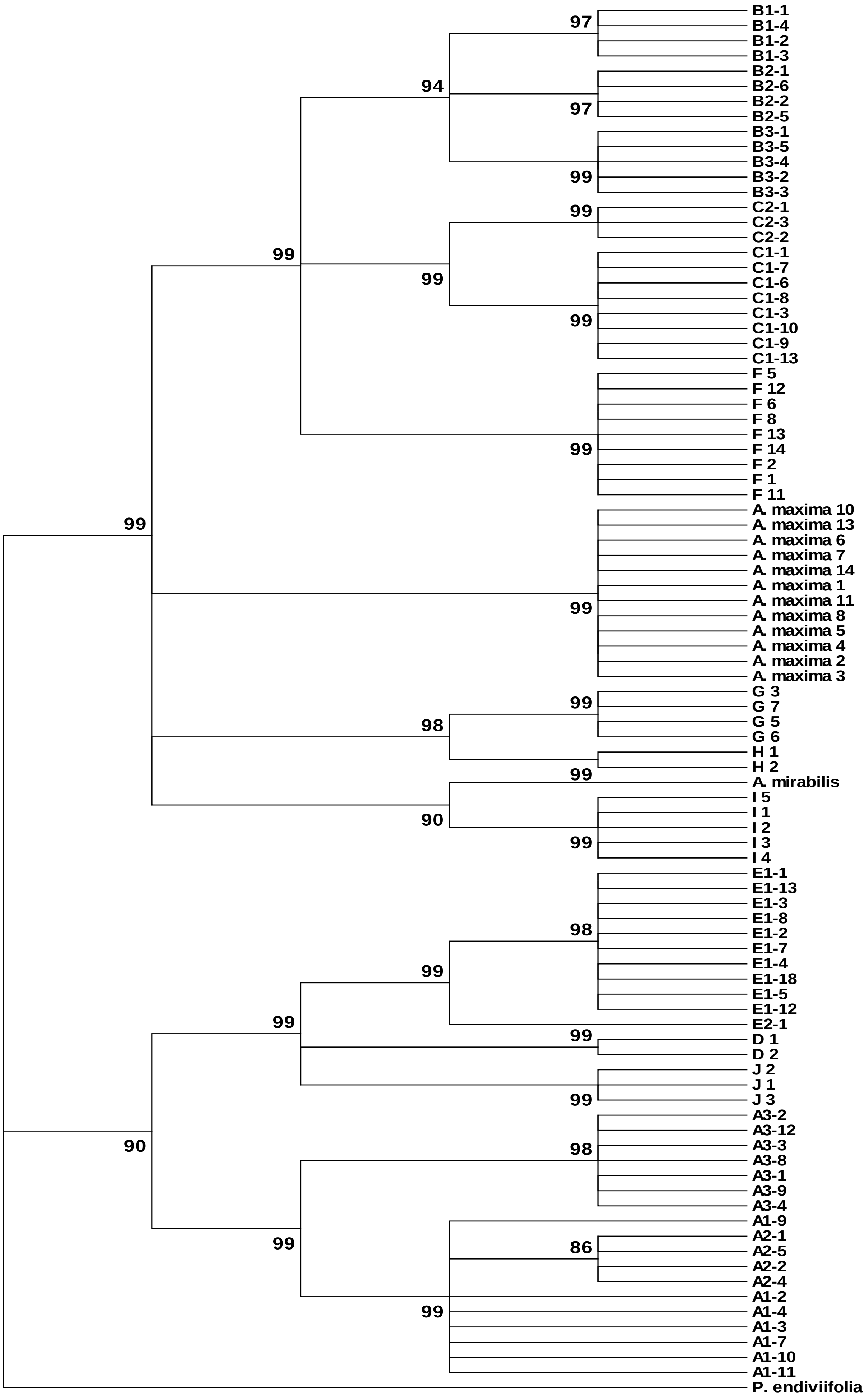

matK+trnH-psbA

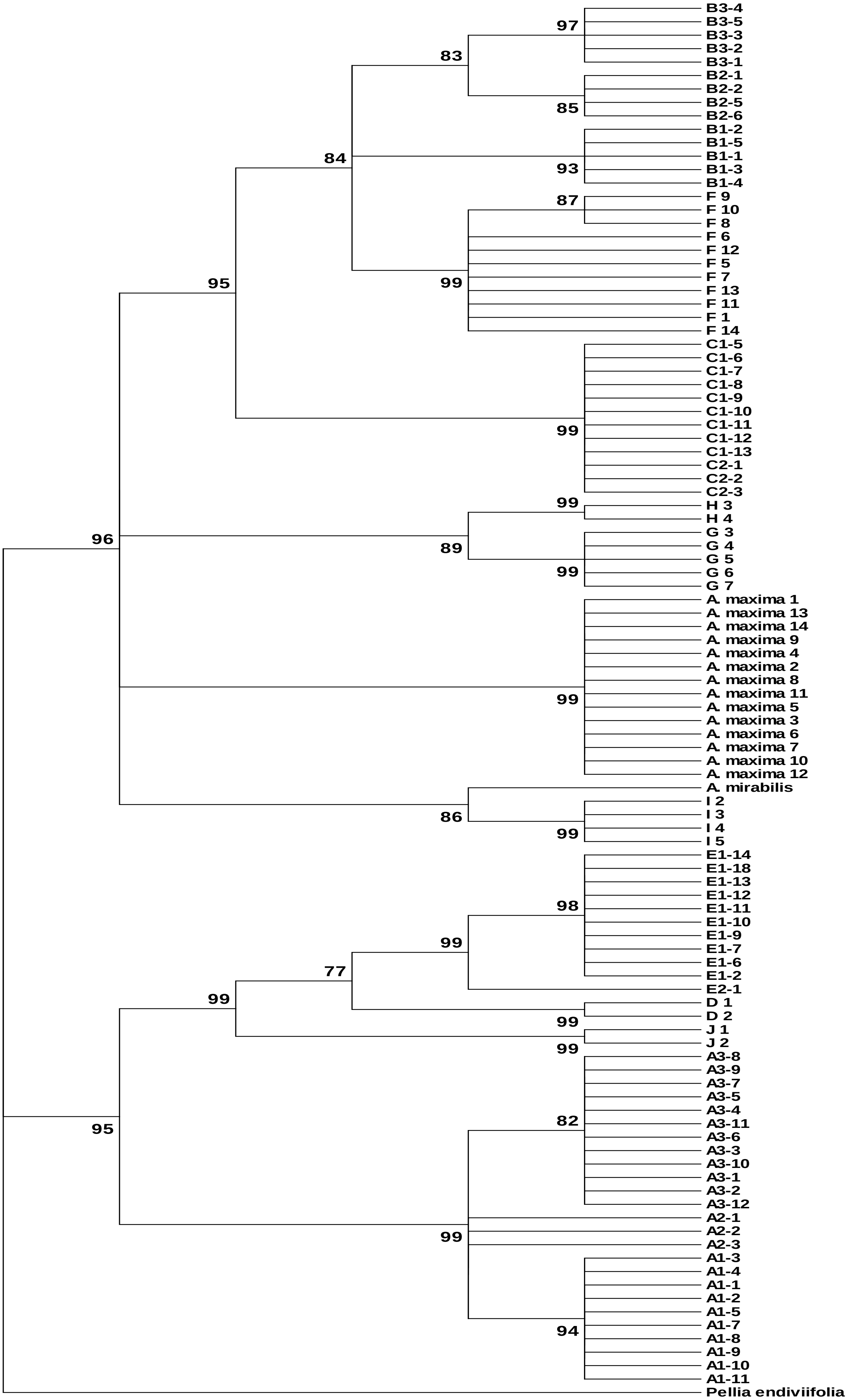
B3-4
B3-5
B3-3
B3-2
B3-1
B2-1
B2-2
B2-5
B2-6
B1-2
B1-5
B1-1
B1-3
B1-4
F 9
F 10
F 8
F 6
F 12
F 5
F 7
F 13
F 11
F 1
F 14
C1-5
C1-6
C1-7
C1-8
C1-9
C1-10
C1-11
C1-12
C1-13
C2-1
C2-2
C2-3
H 3
H 4
G 3
G 4
G 5
G 6
G 7
A. maxima 1
A. maxima 13
A. maxima 14
A. maxima 9
A. maxima 4
A. maxima 2
A. maxima 8
A. maxima 11
A. maxima 5
A. maxima 3
A. maxima 6
A. maxima 7
A. maxima 10
A. maxima 12
A. mirabilis
I 2
I 3
I 4
I 5
E1-14
E1-18
E1-13
E1-12
E1-11
E1-10
E1-9
E1-7
E1-6
E1-2
E2-1
D 1
D 2
J 1
J 2
A3-8
A3-9
A3-7
A3-5
A3-4
A3-11
A3-6
A3-3
A3-10
A3-1
A3-2
A3-12
A2-1
A2-2
A2-3
A1-3
A1-4
A1-1
A1-2
A1-5
A1-7
A1-8
A1-9
A1-10
A1-11
Pellia endiviifolia
97
83
85
84
93
87
95
99
99
96
99
89
99
99
86
99
98
99
77
99
99
99
82
95
99
94

rbcL+trnL-F

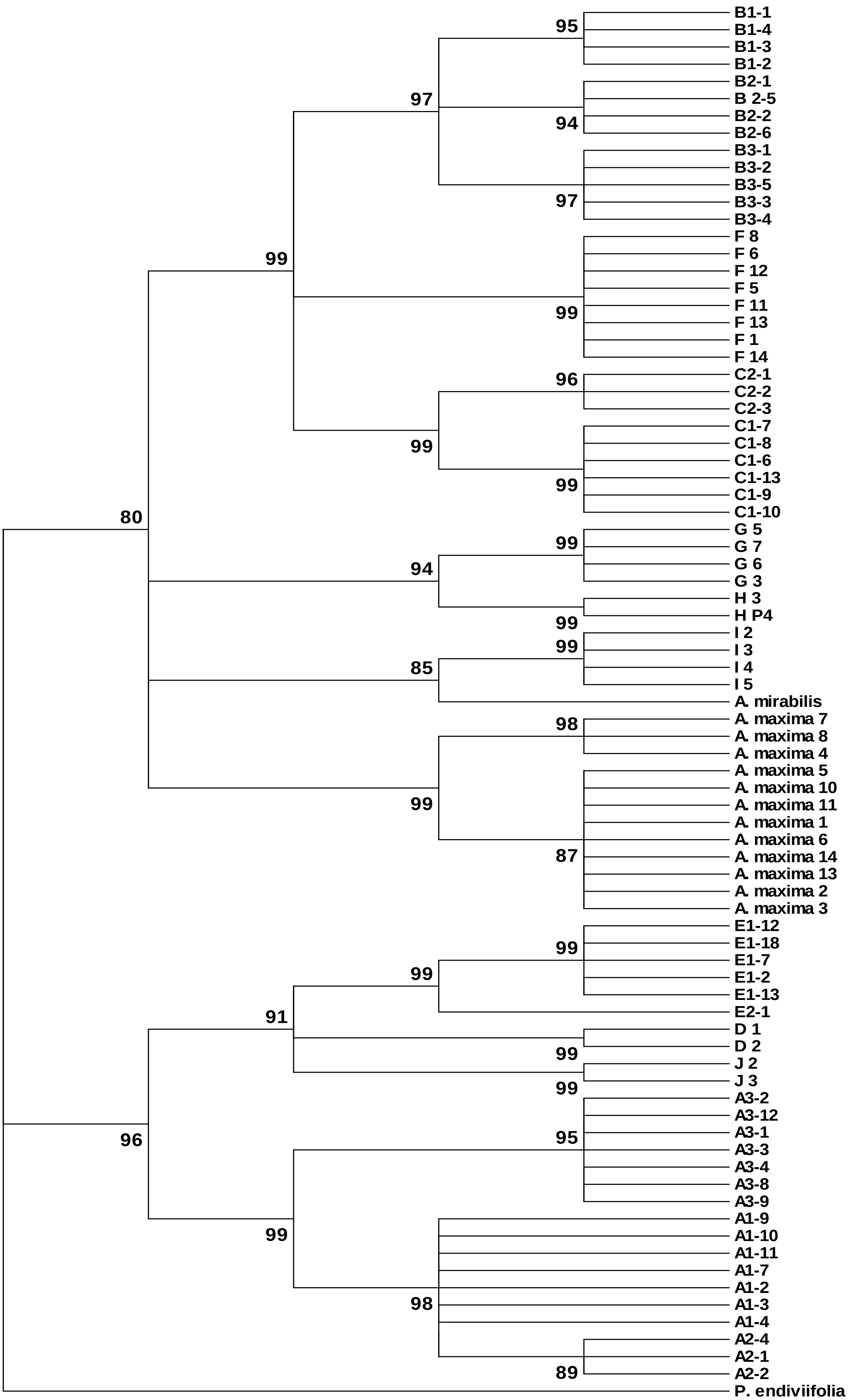

rbcL+trnH-psbA

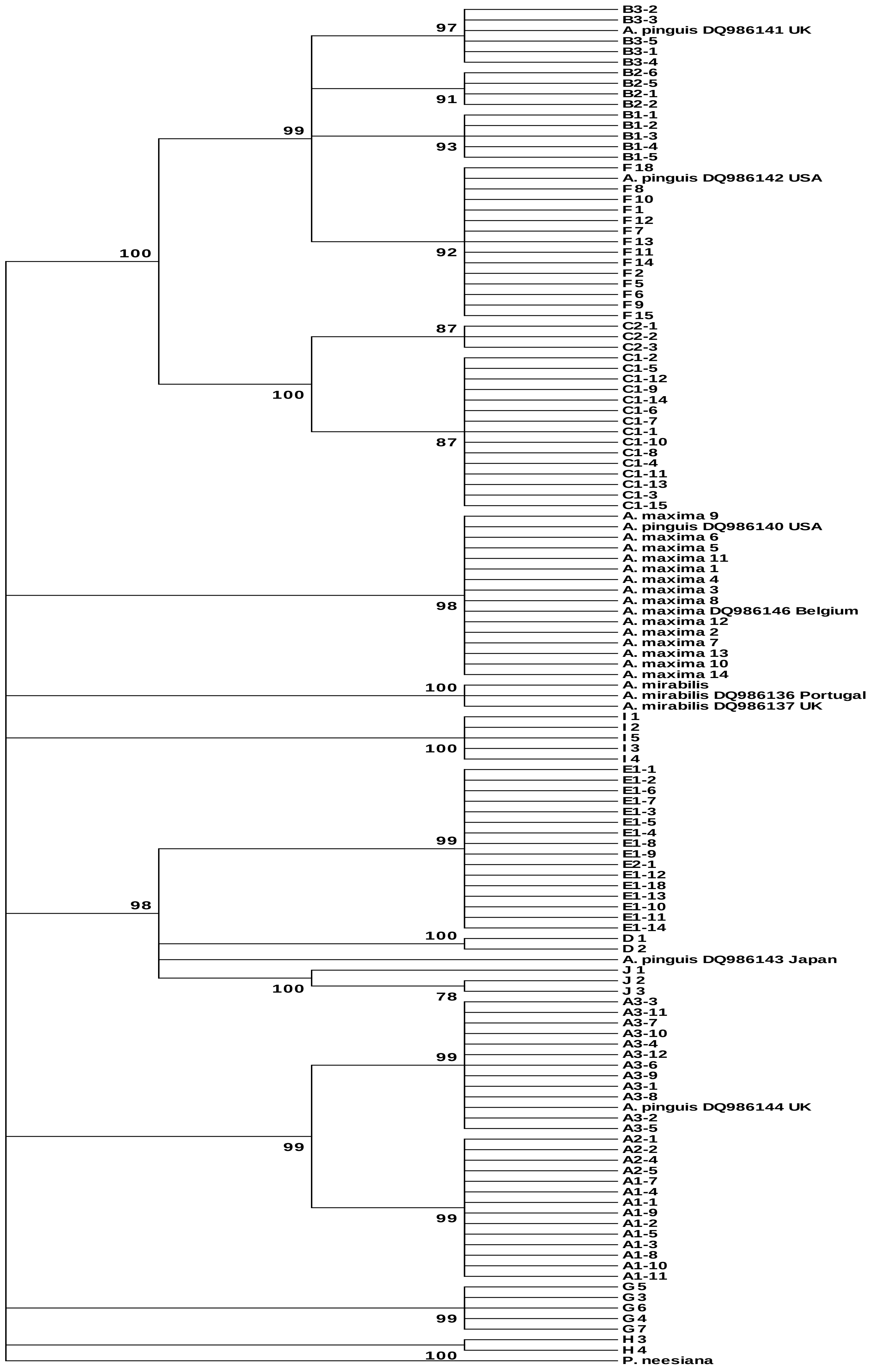
B3-2
B3-3
A. pinguis DQ986141 UK
B3-5
B3-1
B3-4
B2-6
B2-5
B2-1
B2-2
B1-1
B1-2
B1-3
B1-4
B1-5
F18
A. pinguis DQ986142 USA
F8
F10
F1
F12
F7
F13
F11
F14
F2
F5
F6
F9
F15
C2-1
C2-2
C2-3
C1-2
C1-5
C1-12
C1-9
C1-14
C1-6
C1-7
C1-1
C1-10
C1-8
C1-4
C1-11
C1-13
C1-3
C1-15
A. maxima 9
A. pinguis DQ986140 USA
A. maxima 6
A. maxima 5
A. maxima 11
A. maxima 1
A. maxima 4
A. maxima 3
A. maxima 8
A. maxima DQ986146 Belgium
A. maxima 12
A. maxima 2
A. maxima 7
A. maxima 13
A. maxima 10
A. maxima 14
A. mirabilis
A. mirabilis DQ986136 Portugal
A. mirabilis DQ986137 UK
I 1
I 2
I 5
I 3
I 4
E1-1
E1-2
E1-6
E1-7
E1-3
E1-5
E1-4
E1-8
E1-9
E2-1
E1-12
E1-18
E1-13
E1-10
E1-11
E1-14
D 1
D 2
A. pinguis DQ986143 Japan
J 1
J 2
J 3
A3-3
A3-11
A3-7
A3-10
A3-4
A3-12
A3-6
A3-9
A3-1
A3-8
A. pinguis DQ986144 UK
A3-2
A3-5
A2-1
A2-2
A2-4
A2-5
A1-7
A1-4
A1-1
A1-9
A1-2
A1-5
A1-3
A1-8
A1-10
A1-11
G 5
G 3
G 6
G 4
G 7
H 3
H 4
P. neesiana
97
91
99
93
92
100
87
100
87
98
100
100
99
98
100
100
78
99
99
99
99
100

ITS1

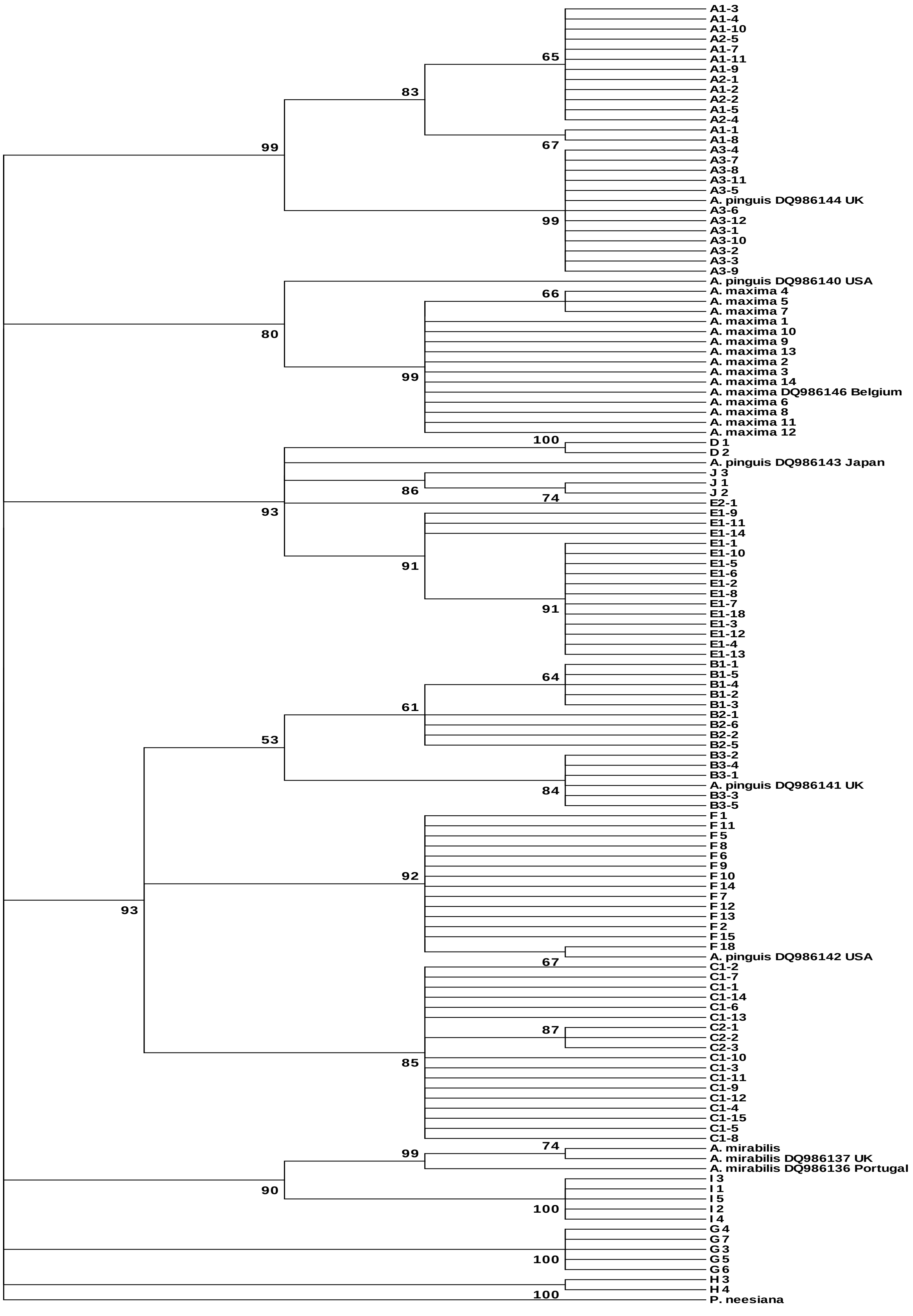
A1-3
A1-4
A1-10
A2-5
A1-7
A1-11
A1-9
A2-1
A1-2
A2-2
A1-5
A2-4
A1-1
A1-8
A3-4
A3-7
A3-8
A3-11
A3-5
A. pinguis DQ986144 UK
A3-6
A3-12
A3-1
A3-10
A3-2
A3-3
A3-9
A. pinguis DQ986140 USA
A. maxima 4
A. maxima 5
A. maxima 7
A. maxima 1
A. maxima 10
A. maxima 9
A. maxima 13
A. maxima 2
A. maxima 3
A. maxima 14
A. maxima DQ986146 Belgium
A. maxima 6
A. maxima 8
A. maxima 11
A. maxima 12
D 1
D 2
A. pinguis DQ986143 Japan
J 3
J 1
J 2
E2-1
E1-9
E1-11
E1-14
E1-1
E1-10
E1-5
E1-6
E1-2
E1-8
E1-7
E1-18
E1-3
E1-12
E1-4
E1-13
B1-1
B1-5
B1-4
B1-2
B1-3
B2-1
B2-6
B2-2
B2-5
B3-2
B3-4
B3-1
A. pinguis DQ986141 UK
B3-3
B3-5
F 1
F 11
F 5
F 8
F 6
F 9
F 10
F 14
F 7
F 12
F 13
F 2
F 15
F 18
A. pinguis DQ986142 USA
C1-2
C1-7
C1-1
C1-14
C1-6
C1-13
C2-1
C2-2
C2-3
C1-10
C1-3
C1-11
C1-9
C1-12
C1-4
C1-15
C1-5
C1-8
A. mirabilis
A. mirabilis DQ986137 UK
A. mirabilis DQ986136 Portugal
I 3
I 1
I 5
I 2
I 4
G 4
G 7
G 3
G 5
G 6
H 3
H 4
P. neesiana
65
83
99
67
99
66
80
99
100
86
74
93
91
91
64
61
53
84
92
93
67
87
85
99
74
90
100
100
100

ITS2

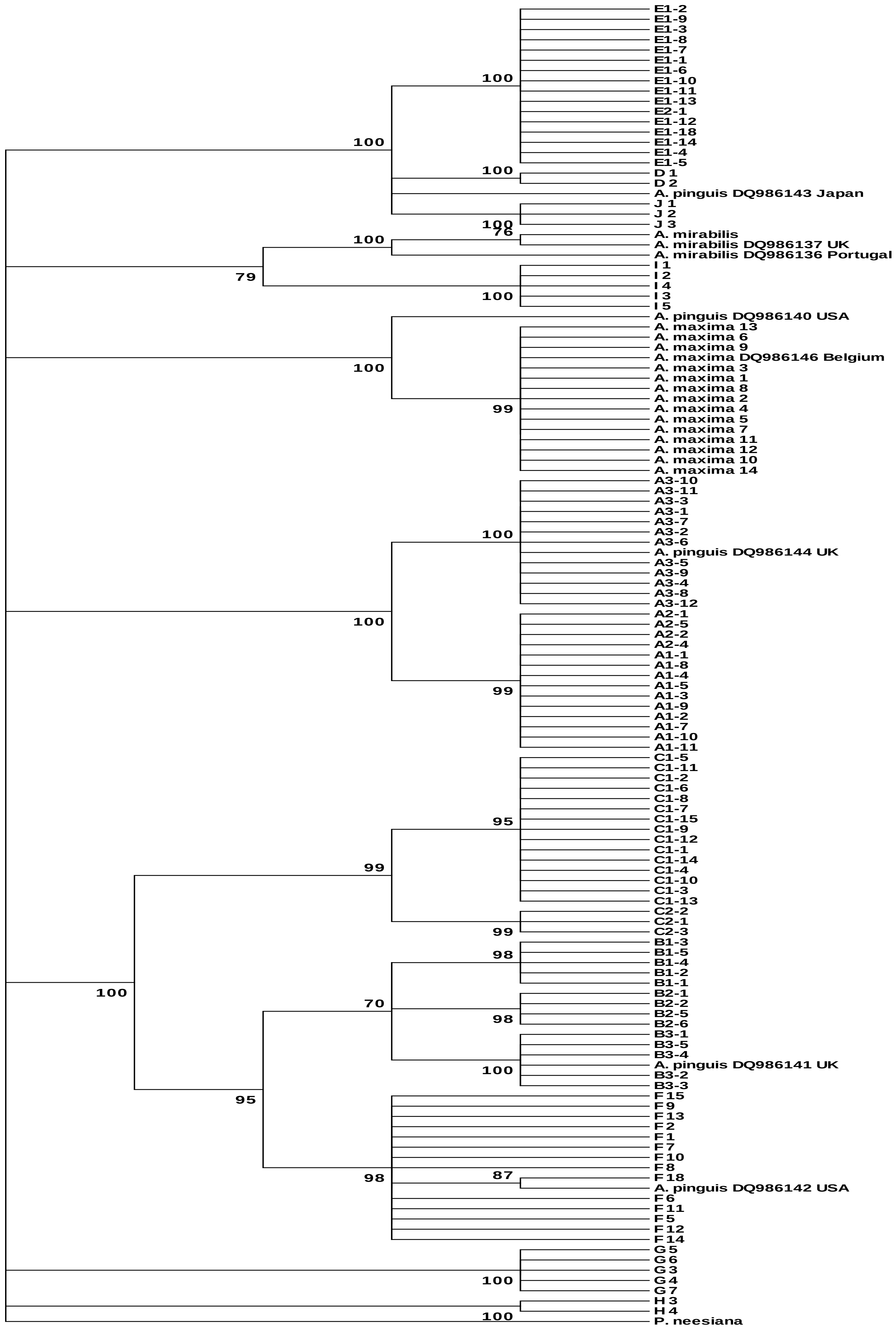
E1-2
E1-9
E1-3
E1-8
E1-7
E1-1
E1-6
E1-10
E1-11
E1-13
E2-1
E1-12
E1-18
E1-14
E1-4
E1-5
D 1
D 2
A. pinguis DQ986143 Japan
J 1
J 2
J 3
A. mirabilis
A. mirabilis DQ986137 UK
A. mirabilis DQ986136 Portugal
I 1
I 2
I 4
I 3
I 5
A. pinguis DQ986140 USA
A. maxima 13
A. maxima 6
A. maxima 9
A. maxima DQ986146 Belgium
A. maxima 3
A. maxima 1
A. maxima 8
A. maxima 2
A. maxima 4
A. maxima 5
A. maxima 7
A. maxima 11
A. maxima 12
A. maxima 10
A. maxima 14
A3-10
A3-11
A3-3
A3-1
A3-7
A3-2
A3-6
A. pinguis DQ986144 UK
A3-5
A3-9
A3-4
A3-8
A3-12
A2-1
A2-5
A2-2
A2-4
A1-1
A1-8
A1-4
A1-5
A1-3
A1-9
A1-2
A1-7
A1-10
A1-11
C1-5
C1-11
C1-2
C1-6
C1-8
C1-7
C1-15
C1-9
C1-12
C1-1
C1-14
C1-4
C1-10
C1-3
C1-13
C2-2
C2-1
C2-3
B1-3
B1-5
B1-4
B1-2
B1-1
B2-1
B2-2
B2-5
B2-6
B3-1
B3-5
B3-4
A. pinguis DQ986141 UK
B3-2
B3-3
F 15
F 9
F 13
F 2
F 1
F 7
F 10
F 8
F 18
A. pinguis DQ986142 USA
F 6
F 11
F 5
F 12
F 14
G 5
G 6
G 3
G 4
G 7
H 3
H 4
P. neesiana
100
100
100
100
76
100
79
100
100
99
100
100
100
99
95
99
99
98
100
70
98
100
95
98
87
100
100

ITS
